# The water quality of the Upper Citarum: Applying the overall index of pollution, Said-WQI, and pollution index methods

**DOI:** 10.1016/j.heliyon.2025.e41690

**Published:** 2025-01-08

**Authors:** Mariana Marselina, Nurul Aulia Rahmi, Siti Ai Nurhayati

**Affiliations:** Faculty of Civil and Environmental Engineering, Bandung Institute of Technology, Bandung, Indonesia

**Keywords:** OIP, Pollution index (PI), Said-WQI, Upstream Citarum, Water quality index

## Abstract

Water is a crucial natural resource, integral to sustaining human life and environmental equilibrium. However, water quality remains a critical issue issue due to prevailing contamination. While river water is a primary source of raw drinking water, much of it, such as Indonesia's Citarum River in West Java, has been polluted. While studies in Indonesia using the Water Quality Index (WQI) are limited, various measurement methods have been developed globally to evaluate water quality. This research compares three methods – the Overall Index of Pollution (OIP), Said-WQI method, and Pollution Index (PI) – to assess the water quality of Upstream Citarum River. The research utilized both primary and secondary data derived from Citarum River samples. Additionally, analytical tools including Microsoft Excel, Geographic Information System (GIS), and SPSS, were used for data processing, rainfall analysis, and statistical testing, respectively. Results from four river-monitoring points indicated average values for key parameters such as biochemical oxygen demand, dissolved oxygen (DO), and total and fecal coliform, falling below established quality standards. WQI measurements revealed variable degrees of pollution in the Upstream Citarum River according to the method used. The OIP and Said-WQI methods categorized the river's status as ranging from ‘good’ to ‘poor’, while the PI method classified it from ‘mildly polluted’ to ‘severely polluted’. Seasonal analysis of wet and dry months using secondary data revealed OIP index values ranging from 3.71 to 11.20, classifying water quality as "poor" to "moderate". The Said-WQI method yielded values between 0.67 and 2.34, indicating "poor" to "good" quality, while the PI method produced values from 4.15 to 8.13, denoting “moderately polluted” to “heavily polluted” conditions. Spatial analysis showed that upstream conditions were better than downstream conditions. The OIP index ranged from 3.71 upstream to 11.20 downstream with a classification of “poor” to “good”. The Said-WQI index ranged from 0.67 upstream to 2.34 downstream, classified as – “poor” to “good”. Similarly, the PI index ranged from 4.15 upstream to 8.13 downstream, indicating “moderately polluted” to “severely polluted” conditions. These findings from secondary data were corroborated by primary field sampling results. This study successfully evaluated the water quality of the Upper Citarum River by comparing measurement data with established standards across various locations and time periods. Furthermore, it conducted a comparative analysis of the differences between the three indices, focusing on parameters, sub-index transformations, weighting, and aggregation processes.

## Highlights

• The water quality index is recognized as an effective tool for assessing the impact of water quality and is useful for conveying water quality information to policymakers and the public.
• Water quality status was calculated using three methods: the Overall Index of Pollution-Water Quality Index, Said-Water Quality Index, and Pollution Index.
• Water quality analysis can employ both primary and secondary datasets.
• According to the OIP and Said-WQI calculation methods, the water quality status of the Upper Citarum River varies from ‘Good’ to ‘Moderate’ and ‘Poor’.

## 1 Introduction

Water is a vital resource central to sustaining life, ecosystems, and human activities [1,2]. Beyond its critical ecological functions, water serves as a cornerstone for economic development and various industries [3]. Ensuring both the availability and quality of water is, therefore, essential. Among the various sources of water, rivers are particularly significant due to their accessibility and abundance [4].

However, rising population, urbanization, and socio-economic development have strained water resources. Additional stress arises from mining, agricultural practices, and land-use changes, all of which contribute to water quality degradation. This situation is exacerbated by the unregulated disposal of untreated wastewater containing organic and inorganic pollutants into water bodies [[5], [6], [7]].

Rivers, being susceptible both to human-induced and natural contamination, are most impacted. Their water quality is vulnerable to factors like rainfall, weather conditions, and sediment transport dynamics [7]. Given these challenges, studying water quality has emerged as a crucial environmental research area in the 21st century. The need for such research is accentuated by the extensive use of water in various human activities such as industry, agriculture, construction, and domestic consumption, all of which necessitate a certain degree of water quality [8,9].

The regular monitoring and maintenance of water quality are imperative to preserving a balanced environment [5,7,8]. It is also a matter of public health issue, as poor water quality can adversely affect both the population and the environment. If left unchecked, declining water quality will diminish the functionality, productivity, and capacity of water resources, ultimately reducing natural wealth [2,10].

Research comparing water quality on Java and Kalimantan Islands revealed that Java experiences significantly a higher level of water pollution. The study indicated an average coliform contamination rate of 61.42 % in Java Island's water sources [11]. Furthermore, a 2019 appraisal of Indonesia's rivers classified them as predominantly polluted. Out of 98 rivers assessed, 54 were categorized as slightly polluted, six ranged from lightly to moderately polluted, and 38 were from moderately to heavily polluted.

Citarum River, spanning roughly 300 km, is the longest river in West Java Province. This strategic and vital waterway supplies water to 80 % of the residents in the watershed area of the Special Capital Region of Jakarta.

In 2018, the West Java Provincial Government reported that the Citarum River's water quality status remained heavily polluted, with a Water Quality Index (WQI) value of 33.43. By 2019, this value had increased to 40.20 [[12], [13], [14]]. However, a separate 2019 study found that the Citarum River's pollution status had improved from “heavily polluted” to “moderately polluted” [15].

In Indonesia, Water Quality Index (WQI) studies are infrequently conducted using methods other than the Pollution Index (PI) established by the Regulation of the Minister of Environment of the Republic of Indonesia in 2003 [4]. This contrasts with practices in many other countries, where a variety of WQI techniques are employed. However, even when applied to the same surface water, different WQI methods often yield varying results [16]. For instance, a 2022 study using the National Sanitation Foundation WQI (NSFWQI), the Canadian Council of Ministers of the Environment WQI (CCMEWQI), and the Oregon WQI (OWQI) methods produced differing water quality assessments for the Citarum River [17]. These discrepancies arise from variations in the number of parameters used, classification scales, assigned weights, and the final interpretation of the WQI.

In another hand, Mokarram et al. [18] have conducted a study to evaluate the quality of the Kor River in Southern Iran, identifying the main sources of contaminations discharged to the river by neighboring factories. The Water Quality Index and Heavy Metal Evaluation Index were used to determine contamination levels at each station. Stations with water quality indices of 0.9 ± 5.0 and 87.3 ± 2.0 represented the highest and the lowest contamination levels, respectively, corresponding to very poor (undrinkable) and very good water qualities. Stations with poor water quality were also found to be highly polluted according to the Heavy Metal Evaluation Index, showing high concentrations of arsenic and cadmium. Consequently, no universally accepted method exists for calculating water quality management indices [19].

This study explores three methods for measuring the WQI: the Overall Index of Pollution (OIP), the WQI developed by Said (Said-WQI), and the Pollution Index (PI), which is frequently used in Indonesia. The OIP method is recognized as an effective tool for assessing the impact of water quality changes along rivers and for conveying water quality information to policymakers and the public. This tool assists in addressing issues associated with local and regional surface water quality [20]. The Said-WQI method, developed by Said, simplifies the assessment process by reducing the number of variables and using logarithmic aggregation procedures [21]. This strategy is recognized for its simplicity, speed, and the lack of a need for standardizing water quality variables or computing sub-indices. It also reduces the quantity of water quality variables necessary to assess water quality conditions [22].

This study aimed to achieve three key objectives. Firstly, it endeavored to evaluate the water quality of the Upper Citarum River by comparing measurement data with established standards at various points and across different time periods. Secondly, it sought to ascertain the WQI of the Upper Citarum River using three calculation methods: OIP, Said-WQI, and PI within a similar spatial and temporal context as the first objective. Thirdly, it aimed to compare the effectiveness of the three aforementioned methods based on the gathered research data.

Although the assessment of the water quality status of the Upper Citarum River has been carried out using several types of index calculation methods, no prior study has calculated the OIP and Said WQI indices. Both of these indices are commonly used in developing countries such as Indonesia. Therefore, calculating these indices is valuable for providing insights into water quality assessment in Indonesian rivers, particularly in terms of parameter selection and the weight assigned to each parameter.

## 2 Materials and methods

### 2.1 Study location and data collection

**Fig. 1:**
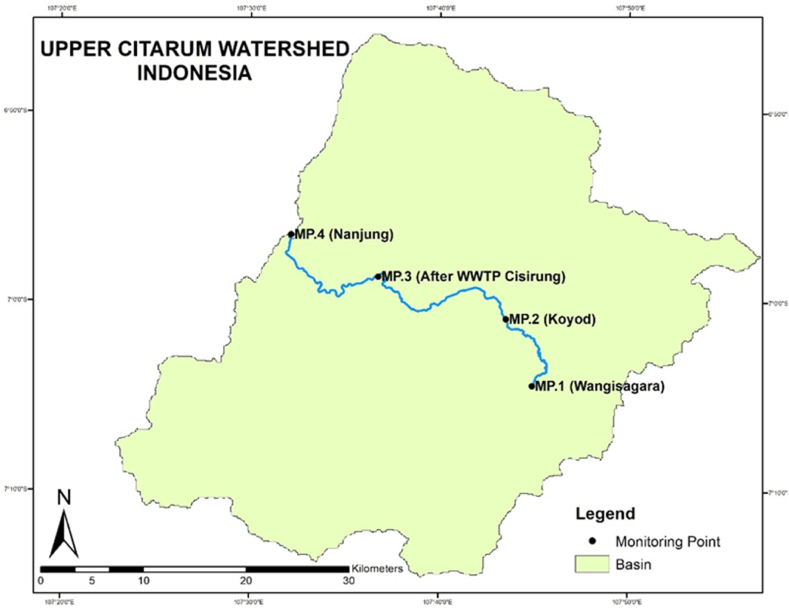
Map of the monitoring point.

This study was conducted in the Upper Citarum River and involved sampling at four monitoring locations: Wangisagara (MP.1), Koyod (MP.2), after-Wastewater Treatment Plant (WWTP) Cisirung (MP.3), and Nanjung (MP.4) which can be seen in Fig. 1. The selection of these monitoring points aligns with those utilized by the West Java Provincial Environmental Agency. To gather relevant data, both primary and secondary sources were utilized. Secondary data consisted of water quality records from the Citarum River monitoring points spanning 2013 to 2022, obtained from the Regional Environmental Management Agency of West Java Province. Additionally, rainfall data were sourced from the Citarum Major River Basin Organization (BBWS) and the Bandung Meteorological, Climatological, and Geophysical Agency (BMKG).

Primary data were collected twice to represent water quality during Indonesia's two distinct seasons. The first collection took place in February to reflect the wet season, while the second took place in June, representing the dry season. Water quality parameters were tested using two methods, in situ and ex situ. In-situ tests, conducted directly on-site, measured parameters that are prone to rapid changes, such as pH, temperature, and dissolved oxygen (DO), in compliance with the Indonesian National Standard (SNI) 6989.57: 2008 [23]. Ex-situ tests were performed in a laboratory setting to measure parameters such as Biological Oxygen Demand (BOD), Total Dissolved Solids (TDS), nitrate (NO_3_), turbidity, total coliform, fecal coliform, electrical conductivity, and total phosphate. These tests also adhered to the Indonesian National Standard (SNI).

### 2.2 Improvement and grouping of water quality data

This study's data set was extensive, necessitating standardization and outlier correction. Data preparation began with the removal of incomplete subsets containing empty, zero, or censored values. Eliminating these entries was crucial to prevent calculation errors and ensure the precise execution of the WQI [24].

Given the varied sizes and units of the water quality data, standardization was required to achieve uniformity. This was accomplished using the Z-score formula, as illustrated in Equation (1) [25]. Outliers, or data points significantly different from the rest, were identified through an outlier test. These outliers could result from inaccurate data entry, sampling errors, or specific extreme conditions. Standardized data with Z-score values smaller than −3 or larger than 3 were categorized as outliers [26].

(1) $$Z_{i}=\frac{\left(X_{i}-X_{r}\right)}{S}$$

where Z_i_ is the standardized data value for data point *i*; X_r_ is the mean of the data; X_i_ is data point i; and S is the standard deviation.

To streamline processing and analysis, particularly for secondary data, the data were categorized into four groups: 1) data per monitoring point, representing the mean of all years of monitoring for each point; 2) data per monitoring year, signifying the average data for each monitoring year across all points; 3) data from wet and dry months, or the average data from all months classified as either wet or dry; 4) data from wet and dry years, or the mean results from years identified as such. The distinctions between wet and dry months were determined by analyzing rainfall data from four stations (Rancaekek, Kertasari, Cidurian, and Hantap) for the period from 2013 to 2021.

The average rainfall was determined using the Polygon Thiessen method. This technique assumes that rainfall measured at a station represents the surrounding area influenced by that station post. It assigns a weight to each station proportional to its area of influence, as calculated using QGIS software. The formula is provided in Equation (2) [27].

(2) $$P=\frac{A_{1}P_{1}+A_{2}P_{2}+\ldots +A_{n}P_{n}}{A_{1}+A_{2}+\ldots +A_{n}}$$

where P is the area-averaged rainfall; P1, P2, …, Pn are the rainfall measurements at stations 1, 2, … n; and A1, A2, … An are the areas representing stations 1, 2, … n.

Rainfall data were averaged monthly and annually. Subsequently, the data were ranked from highest to lowest based on Markov theory to compute the probability (P). Finally, this was segmented into descriptors such as wet and dry months, as well as wet and dry years.

### 2.3 Water quality index calculation method

Three different methods were used to determine the Water Quality Index in this study: the Overall Index of Pollution (OIP) method, the Said-WQI method, and the PI method. The OIP method was created to streamline and specify water quality data analysis. It employs a mixed parameter selection system, which involves predetermined basic parameters that can be adjusted as needed.

This method evaluates surface water quality by scoring measurements of various elements, such as pH, 5-day biological oxygen demand (BOD5), and total coliform levels. The results are classified into five categories: Excellent (0–1 numerical estimate), Acceptable (1–2), Slightly Polluted (2–4), Polluted (4–8), and Heavily Polluted (8–16) [20,28].

The selection of parameters for this method considers the limited availability of secondary data and ensures the continuity of data measurement [29]. The water quality data are transformed into sub-indices using mathematical equations for each parameter's concentration level as shown in Table 1 [28].

**Table 1:** Transformation Equations for Sub-Indices in the OIP method.

**Parameter**		**Mathematical Equations**
**Turbidites**	≤5	x = 1
	5–10	x = (y/5)
	10–500	x = (y + 43.9)/34.5
**pH**	7	x = 1
	>7	x = exp ((y-7)/1.082)
	<7	x = exp ((7-y)/1.082)
**%DO**	<50	x = exp (-(y – 98.33)/36.067)
	50–100	x = (y-107.58)/14.667
	≥100	x = (y – 79,543)/19,054
**BOD**_**5**_	<2	x = 1
	2–30	x = y/1.5
**TDS**	≤500	x = 1
	500–1500	x = exp ((y-500)/721.5
	1500–3000	x= (y – 1000)/12.5
	3000–6000	x = y/375
**NO**_**3**_	≤20	x = 1
	20–50	x = exp((y-145.16)/76.28)
	50–200	x = y/65
**Koliform Total**	≤50	x = 1
	50–5000	x = ((y/50)^0.301^
	5000–15000	x = ((y/50) – 50)/16,071
	>15000	x = (y/15000) + 16

The Overall Index of Pollution (OIP) is calculated using Equation (3) [28].

(3) $$OIP=\frac{\sum_{i}P_{i}}{n}$$

Where *P*_*i*_ is the PI for the *i-*th parameter, *i* = 1, 2, …, n; and n is the total number of parameters.

The Said-WQI method uses five fixed parameters: dissolved oxygen, total phosphate, fecal coliform (FCol), turbidity (Turb), and specific conductivity (SC). These parameters are preselected and cannot be modified, ensuring a rigid framework. The Said-WQI is scaled from 0 to 3 with the following classifications: Poor (0–1), Remediation Needed (1–2), Acceptable (2–3), and Very Good (3) [21,22]. The index value is calculated using Equation (4) [22].

(4) $$WQI=\log\left[\frac{{\left(DO\right)}^{1.5}}{{\left(3.8\right)}^{TP}{\left(Turb\right)}^{0,15}{\left(15\right)}^{FCol/10000}+{0.14\left(SC\right)}^{0.5}}\right]$$

where DO is the dissolved oxygen (% oxygen saturation); TP is the total phosphates (mg/L); Turb is the turbidity (NTU); FCol is the fecal coliform bacteria (counts/100 ml); SC is the specific conductivity in (MS/cm at 25 °C).

The PI measures relative pollution levels based on authorized water quality parameters. As an open system, the PI's allows for flexibility in selecting parameters and their quantities. This study utilizes available and continuous data aligned with the prescribed water quality standards of Indonesia (Class 2 Water Quality Standards, Government Regulation Number 22 of 2021) [30]. Out of the ten parameters applied in the OIP and Said-WQI methods, eight were selected for the PI method. Electrical conductivity and turbidity were excluded due to the absence of Indonesian quality standards for these parameters. The calculation for the PI method follows the guidelines provided in Appendix II of the Decree of the State Minister of Environment Number 115 of 2003, outlining water quality status guidelines [31].

The parameter's value or concentration is denoted by Ci, while the quality standard is represented as Li. We convert these values into sub-indices by dividing Ci by Li. If the result of Ci/Lij exceeds 1, we recalculate it using Equation (5).

(5) $$\left(C_{i}/L_{ij}\right)new=1+P.\log\left(\frac{C_{i}}{L_{ij}}\right)measurementresults$$

For the concentration of parameters where value decreases, stating that the level of pollution increases, such as DO, a new value (Ci/Lij) is calculated using Equation (6).

(6) $$\left(C_{i}/L_{ij}\right)new=\frac{C_{im}-C_{i}\left(measurement\;results\right)}{C_{im}-L_{ij}}$$

where C_im_ is for DO maximum (used 7 mg/L0; C_i_ is DO concentration (mg/L); and L_ij_ is quality standard (mg/L). Meanwhile, for quality standards of parameters that have a range such as pH, the Equation (7) and Equation (8) are used.

(7) $$\left(C_{i}/L_{ij}\right)new=\frac{\left[C_{i}-\left(L_{ij}\right)average\right]}{\left[\left(L_{ij}\right)minimum-\left(L_{ij}\right)average\right]}\;\text{for}\;C_{i}\;>\;L_{\text{ij}}\;\text{average}$$

(8) $$\left(C_{i}/L_{ij}\right)new=\frac{\left[C_{i}-\left(L_{ij}\right)average\right]}{\left[\left(L_{ij}\right)maximum-\left(L_{ij}\right)average\right]}\;\text{for}\;C_{i}\;<\;L_{\text{ij}}\;\text{average}$$

After converting parameter concentrations value into sub-indices, the aggregation is performed using the Equation (9).

(9) $${PI}_{j}=\sqrt{\frac{\left(C_{i}/L_{ij}\right)M^{2}+\left(C_{i}/L_{ij}\right)R^{2}}{2}}$$

where PI_j_ is the pollution index value; (C_i_/L_ij_)M is (C_i_/L_ij_) is the maximum value; and (C_i_/L_ij_)R is (C_i_/L_ij_) the average value.

Based on the above calculations, the WQI values for the PI method are divided into four classes. 0 ≤ PIj ≤1, classified “Meet quality standards (good condition)”; 1< PIj ≤5, classified “Lightly Polluted”; 5 < PIj ≤10, classified “Moderately Polluted”; PIj >10 is classified as “Heavily Polluted”.

### 2.4 Statistical test calculation method (correlation test)

To simplify interpretation, the index classification results for each method are grouped into categories. This is necessary as each method has unique scales or ranges. The category structure is as follows.

a. Class 1, corresponds to the ‘Very Good’ classification for OIP and Said-WQI methods and the ‘Meet Quality Standards’ classification for PI methods.
b. Class 2, corresponds to the ‘Good’ classification for OIP and Said-WQI methods and the ‘Light Contaminants’ classification for PI methods.
c. Class 3, corresponds to the ‘Medium’ classification for OIP and Said-WQI methods and the ‘Contaminants’ classification for PI methods.
d. Class 4, corresponds to the ‘Poor’ classification for OIP and Said-WQI methods and the ‘Heavy Contaminants’ classification for PI methods.

Statistical analysis is conducted using a correlation test, reflected through a correlation coefficient (r). The correlation coefficient measures the relationship between variables [32]. Its value ranges from −1 to 1. A value of r = −1 signifies a perfect negative correlation, indicating a weak influence of variable X on variable Y. Conversely, r = 1 l indicates a perfect positive correlation, signifying a strong influence of variable X on variable Y [33]. These values serve as the basis for decision-making: if the significance value < 0.05, then the variables are declared correlated; if the significance value > 0.05, the variables are declared uncorrelated.

The degree of relationship used are as follows: (a) Pearson Correlation value 0–2 = No correlation; (b) Pearson Correlation value 0.21–0.4 = Weak correlation; (c) Pearson Correlation value 0.41–0.6 = Medium correlation; (d) Pearson Correlation value 0.61–0.8 = Strong correlation; and (e) Pearson Correlation value 0.81–1 = Perfect correlation.

Correlation tests were conducted for every data group, including monitoring point data, monitoring year data, wet and dry month data, and wet and dry year data. These tests offered a comparison between the outcomes of index computations using both the OIP method and the Said-WQI method against those obtained from the PI method. The purpose was to evaluate the correlation between these methods and the index calculation technique currently used in Indonesia. A strong correlation tends to indicate a higher likelihood of implementing the evaluated method within the Indonesian context.

## 3 Results and discussion

### 3.1 Upper Citarum River water quality

The secondary data analyzed includes the average water quality data from various monitoring points (Wangisagara, Koyod, After Cisirung WWTP, and Nanjung) during the years 2013–2022. Overall, parameters such as TDS, total phosphate, pH, and nitrate fulfilled the quality standards for class 2 water as per Annex VI Government Regulation Number 22 of 2021. However, other parameters, including biochemical oxygen demand, dissolved oxygen (DO), fecal coliform, and total coliform did not meet these quality standards. The primary data, which included average water quality at each monitoring point based on two samplings, also indicated that a few parameters met class 2 water quality standards, like TDS, total phosphate, DO, pH, and nitrate. Conversely, BOD, fecal coliform, and total coliform failed to meet these standards.

**Fig. 2:**
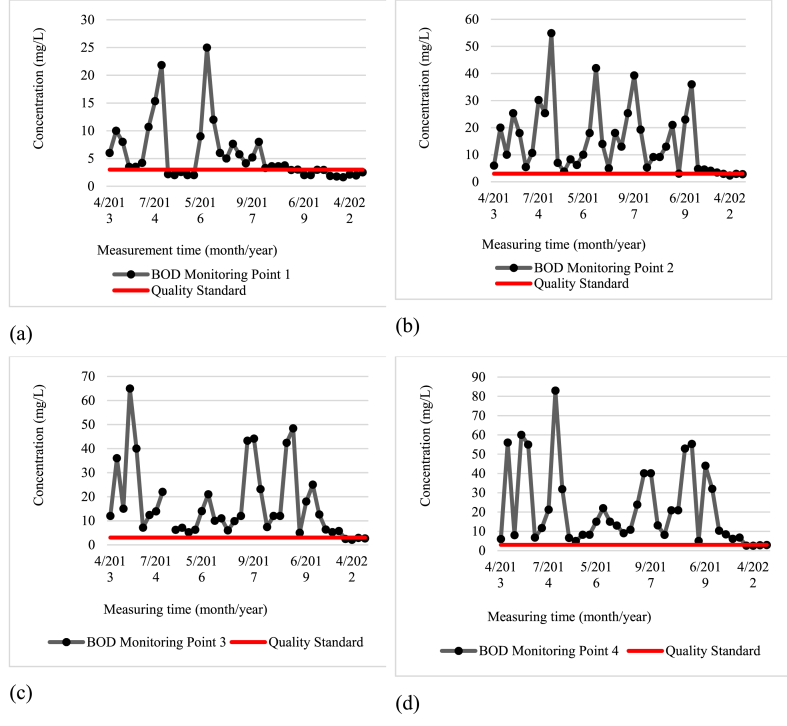
The concentration of the BOD Parameter at each Monitoring Time: (a) Monitoring Point 1 (Wangisagara); (b) Monitoring Point 2 (Koyod Bridge); (c) Monitoring Point 3 (After Cisirung WWTP); and (d) Monitoring Point 4 (Nanjung).

**Fig. 3:**
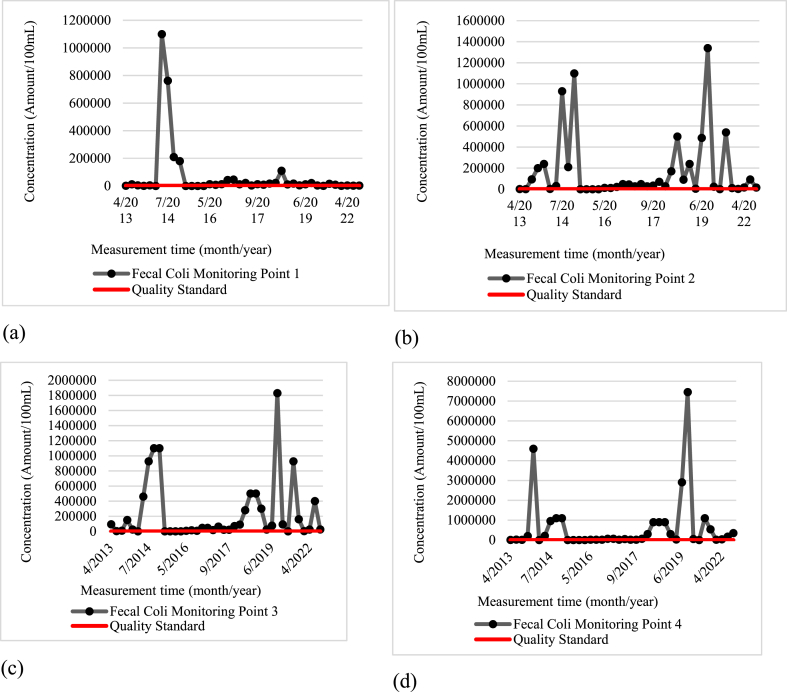
Fecal Coliform Parameter Concentration at each Monitoring Time: (a) Monitoring Point 1 (Wangisagara); (b) Monitoring Point 2 (Koyod Bridge); (c) Monitoring Point 3 (After Cisirung WWTP); and (d) Monitoring Point 4 (Nanjung).

BOD parameters at each monitoring time are illustrated in Fig. 2a for the Wangisagara monitoring point (MP.1), Fig. 2b for the Koyod monitoring point (MP.2), Fig. 2c for the monitoring point after WWTP Cisirung (MP.3), and Fig. 2d for Nanjung monitoring point (MP.4). The fecal coliform parameters compared to the quality standard are illustrated in Fig. 3a for Wangisagara monitoring point (MP.1), Fig. 3b for Koyod monitoring point (MP.2), Fig. 3c for monitoring point after Cisirung WWTP (MP.3), and Fig. 3d for Nanjung monitoring point (MP.4).

**Fig. 4:**
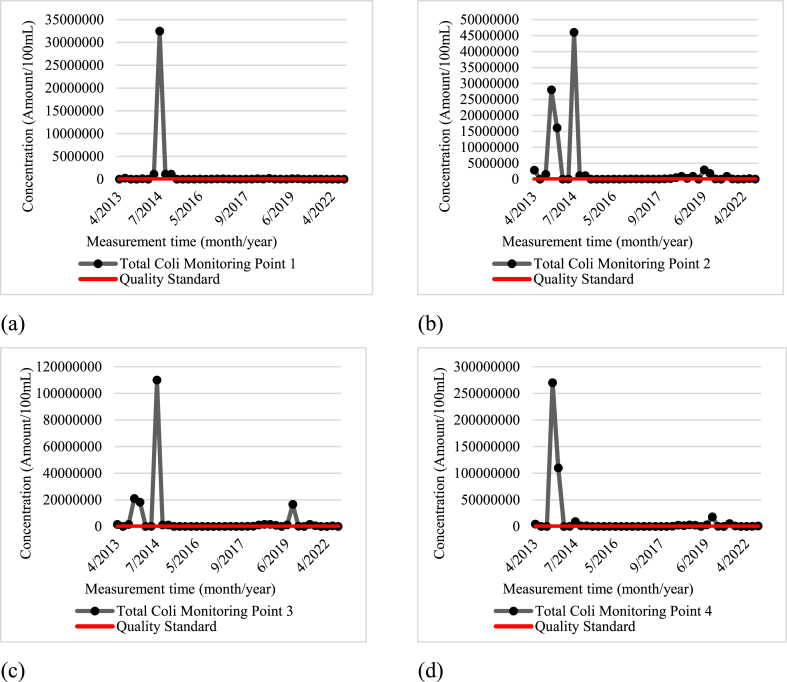
Total Coliform Parameter Concentration at each Monitoring Time: (a) Monitoring Point 1 (Wangisagara); (b) Monitoring Point 2 (Koyod Bridge); (c) Monitoring Point 3 (After Cisirung WWTP); and (d) Monitoring Point 4 (Nanjung).

Fig. 4 shows the concentration of total coliform parameters at each monitoring point compared to the quality standard. The concentration of total coliform at the Wangisagara monitoring point (MP.1) is shown in Fig. 4a. Fig. 4b shows the total coliform concentration at the Koyod monitoring point (MP.2), Fig. 4c shows the total coliform concentration at the After Cisirung WWTP monitoring point (MP.3), and Fig. 4d shows the total coliform concentration at the Nanjung monitoring point (MP.4).

The elevated levels of BOD, fecal coliform, and total coliform in the river are likely due to the presence of waste from nearby industrial processes. Critical land degradation from deforestation, upstream erosion, cow dung, garbage, and industrial waste significantly contribute to the pollution of the Citarum River. This is exacerbated by the high density of industries in the river's catchment area. In 2017, data revealed that the river received 35.5 tons of human waste and 56 tons of livestock waste daily, alongside 20,462 tons of organic and inorganic waste annually. Adding to the severity of the situation, about 90 % of the 3236 textile industries along the river are not equipped with adequate wastewater treatment facilities, leading to the direct discharge of their waste into the river. Consequently, the Citarum River does not meet the quality standards established by the Decree of the Governor of West Java Number 39 Year 2000, as detailed in Refs. [12,13,34]. On the other hand, the high concentrations of BOD, fecal coliform, and total coliform parameters can cause an increase in the incidence of diarrhea or gastrointestinal diseases, thereby significantly reducing the quality of life of the communities around the Citarum River (consequences of elevated levels of BOD, fecal coliform, and total coliform in the river).

### 3.2 Wet month grouping results – dry months and wet years – dry years

Rainfall data was categorized into wet months/dry months and wet years/dry years – utilizing Markov theory. Data was arranged in descending order, and percentages were calculated. Months and years with probabilities under 50 % were classified as wet periods, while those exceeding **50 %** were categorized as dry periods. The results of these calculations are summarized in Table 2 for wet-dry years and Table 3 for wet-dry months.

**Table 2:** Percentage calculation results and data ordering for wet-dry years.

**Annual Average**			
**No**	**Year**	**Amount of rain**	**P (%)**
1	2016	269.42	9.09
2	2015	212.28	18.18
3	2014	212.16	27.27
4	2013	203.02	36.36
5	2022	192.67	45.45
6	2020	190.09	54.55
7	2017	185.66	63.64
8	2021	176.45	72.73
9	2019	152.73	81.82
10	2018	59.67	90.91

**Table 3:** Percentage calculation results and data ordering for wet-dry months.

Monthly Average			
**No**	**Month**	**Amount of rain**	**P (%)**
1	March	314.51	7.69
2	April	313.09	15.38
3	February	282.41	23.08
4	November	281.13	30.77
5	December	266.32	38.46
6	January	188.97	46.15
7	October	170.34	53.85
8	May	161.86	61.54
9	June	87.01	69.23
10	September	65.22	76.92
11	July	65.18	84.62
12	August	28.92	92.31

These include months January, February, March, April, November, and December, as well as the years 2013, 2014, 2015, 2016, and 2022. The dry months are May, June, July, August, September, and October, and the dry years are 2017, 2018, 2019, 2020, and 2021.

### 3.3 Primary data water quality index (WQI) calculation results

#### 3.3.1 Primary data monitoring point

**Fig. 5:**
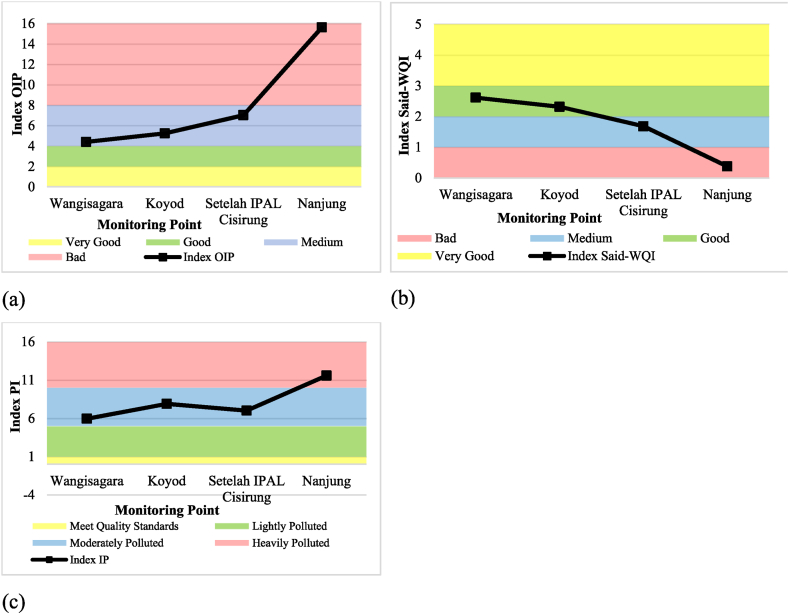
WQI graph for primary data per monitoring point using (a) OIP, (b) Said-WQI, and (c) PI method.

The OIP and Said-WQI calculation results indicate that monitoring point 1 (Wangisagara) has the best water quality. There's a gradual quality decrease from point 2 (Koyod) to point 3 (after WWTP Cisirung), with the lowest quality at point 4 (Nanjung). Contrasting these findings, the PI method identified point 1 (Wangisagara) as having the best water quality, followed by a decrease at point 2 (Koyod). Interestingly, point 3 (after WWTP Cisirung) showed a slight improvement in quality before declining again at point 4 (Nanjung). The calculations from all three WQI models are presented in Table 4 and visualized in Fig. 5a for OIP, Fig. 5b for Said-WQI, and Fig. 5c for PI method.

**Table 4:** Classification of the Upper Citarum River for primary data at monitoring points.

Monitoring Points	Water Quality Index Calculation Method					
	OIP		Said-WQI		PI	
	Index	Classification	Index	Classification	Index	Classification
1	4.40	Medium	2.61	Good	5.94	Moderately Polluted
2	5.24	Medium	2.32	Good	7.89	Moderately Polluted
3	7.01	Medium	1.68	Medium	7.01	Moderately Polluted
4	15.63	Bad	0.37	Bad	11.58	Heavily Polluted

#### 3.3.2 Water quality index based on the wet month and the dry month

**Fig. 6:**
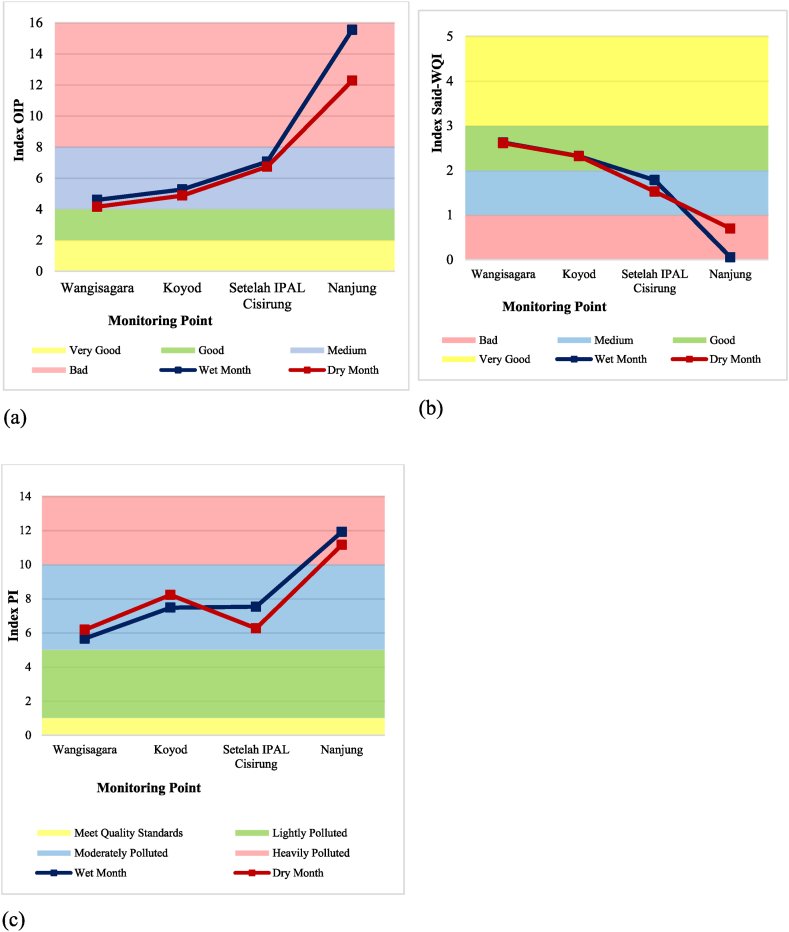
WQI graph for wet month and dry month for primary data using (a) OIP, (b) Said-WQI, and (c) PI method.

The results from the index calculation yield different findings across the three methods used. This variation can be attributed to the differences in parameter concentrations and their values at each monitoring point. The OIP method showed improved water quality during the dry months at all monitoring points. This outcome heavily relies on the lower average concentration of several parameters, such as turbidity, BOD, and total coliform, in dry months. Conversely, the Said-WQI method demonstrated better water quality during the wet months for monitoring points 1, 2, and 3, however, point 4 performed better during the dry months. The PI method indicated better water quality at monitoring points 1 and 2 during wet months, while points 3 and 4 showed improved quality during the dry months. Table 5 provides a summary of the index and classification results from each method used during each season based on primary data. Fig. 6a is a visualization of OIP, Fig. 6b visualization of Said-WQI, and Fig. 6c visualization of the IP method.

**Table 5:** Upper Citarum River classification for wet month and dry month for primary data.

Monitoring Points		Water Quality Index Calculation Method					
		OIP		Said-WQI		PI	
		Index	Classification	Index	Classification	Index	Classification
1	Wet	4.60	Medium	2.623	Good	5.66	Moderately Polluted
	Dry	4.15	Medium	2.608	Good	6.18	Moderately Polluted
2	Wet	5.27	Medium	2.319	Good	7.47	Moderately Polluted
	Dry	4.87	Medium	2.315	Good	8.22	Moderately Polluted
3	Wet	7.05	Medium	1.783	Medium	7.53	Moderately Polluted
	Dry	6.73	Medium	1.525	Medium	6.26	Moderately Polluted
4	Wet	15.54	Bad	0.052	Bad	11.91	Heavily Polluted
	Dry	12.27	Bad	0.698	Bad	11.15	Heavily Polluted

### 3.4 Calculation results of secondary data water quality index

#### 3.4.1 Per secondary data monitoring point

**Fig. 7:**
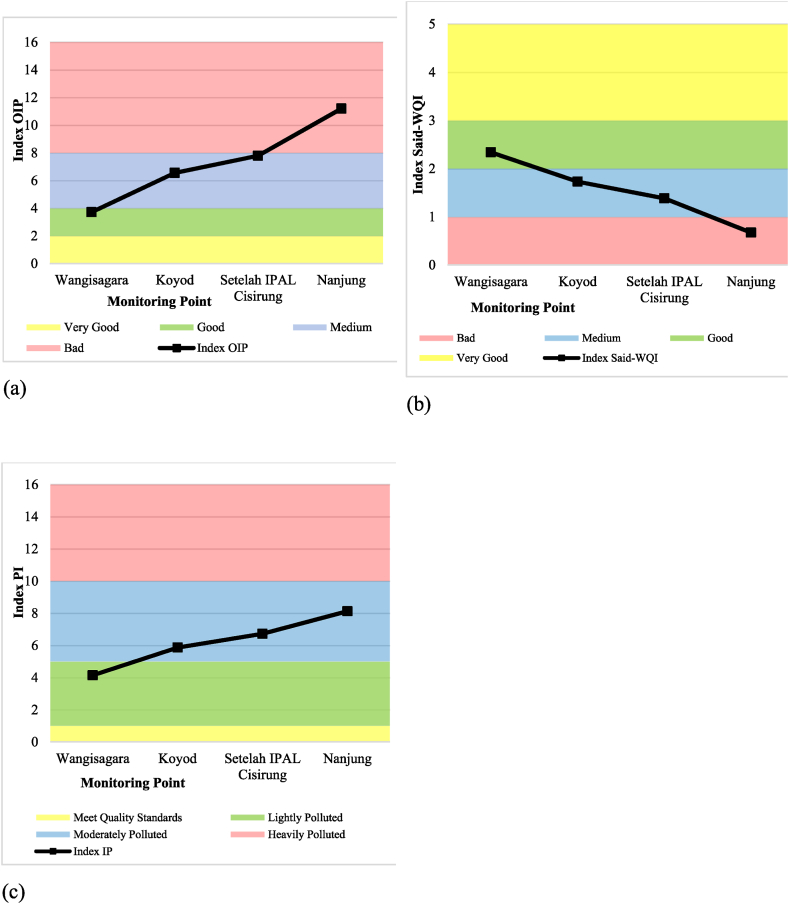
WQI graph for secondary data per monitoring point using (a) OIP, (b) Said-WQI, and (c) PI method.

The OIP and PI methods display improved water quality results as the index values decrease, while the Said-WQI method indicates superior water quality with an increasing index value. The optimal water quality, given the results of these three methods, is located at monitoring point 1 (Wangisagara). The quality decreases at the second monitoring point (Koyod), continues at monitoring point 3 (after WWTP Cisirung), and the poorest water quality is detected at monitoring point 4 (Nanjung). Table 6 provides the index and classification summary using the three methods, Fig. 7a graphically presents the calculation results of the OIP method, Fig. 7b presents the calculation results of Said-WQI, and Fig. 7c graphically presents the results of the IP method.

**Table 6:** Classification of the Upper Citarum River for secondary data at monitoring points.

Monitoring Points	Water Quality Index Calculation Method					
	OIP		Said-WQI		PI	
	Index	Classification	Index	Classification	Index	Classification
1	3.71	Good	2.34	Good	4.15	Lightly Polluted
2	6.55	Medium	1.73	Medium	5.87	Moderately Polluted
3	7.79	Medium	1.38	Medium	6.72	Moderately Polluted
4	11.20	Poor	0.67	Bad	8.13	Moderately Polluted

Water quality tends to deteriorate as the distance from the monitoring point to the upstream source increases. Several factors influence this trend. At point 1, for instance, water quality could be affected by nearby livestock activities. At point 2, the surrounding textile industry could be the primary factor in changes to water quality. Point 3 is a residential area that also serves as a central point for receiving domestic and industrial waste from numerous locations, including Bandung City, Bandung Regency, South Bandung, and Cisirung combined WWTP. Such waste has the potential to trigger changes in the water quality at this point. Lastly, the many industries and settlements in proximity to point 4 might be creating changes in the water quality [35].

#### 3.4.2 Per year secondary data monitoring

The Water Quality Index calculations revealed consistent results for both the OIP and PI methods, while the Said-WQI method showed slight deviations. From 2013 to 2015, water quality improved but declined from 2016 to 2019. After a slight improvement in 2020, it worsened again in 2021 before rising in 2022. The Said-WQI method, however, deviated slightly. Water quality fell between 2013 and 2014, improved in 2015, and then declined again from 2016 to 2019. Similar to the other methods, it showed improvement in 2020, followed by a drop in 2021 and a subsequent increase in 2022. Table 7 summarizes the WQI classifications for each method over the monitoring years.

**Table 7:** Classification of the Upper Citarum River for secondary data per year of monitoring.

Monitoring Year	Water Quality Index Calculation Method					
	OIP		Said-WQI		PI	
	Index	Classification	Index	Classification	Index	Moderately Polluted
2013	15.75	Bad	1.65	Medium	9.30	Moderately Polluted
2014	10.74	Bad	1.06	Medium	8.04	Lightly Polluted
2015	2.06	Good	2.03	Good	1.70	Lightly Polluted
2016	3.64	Good	1.86	Medium	3.13	Lightly Polluted
2017	4.38	Medium	1.70	Medium	3.71	Lightly Polluted
2018	6.01	Medium	1.58	Medium	4.29	Moderately Polluted
2019	7.17	Medium	0.01	Bad	6.46	Lightly Polluted
2020	2.52	Good	2.21	Good	1.97	Moderately Polluted
2021	4.38	Medium	1.80	Medium	3.96	Lightly Polluted
2022	2.52	Good	2.09	Good	1.53	Lightly Polluted

The variation in water quality classification outcomes across different calculation methods can be attributed to differences in the parameters considered. The OIP method identified the highest water quality in 2015, largely due to low turbidity parameters substantially below the set quality standards. This was further influenced by the BOD parameter, which was the third-lowest after the BOD concentrations in 2021 and 2022. On the other hand, the poorest water quality was observed in 2013, primarily affected by high turbidity parameters, second only to those in 2014. Additionally, the nitrate parameters, which ranked second-highest after the nitrate concentration in 2022, also played a role.

The Said-WQI method's calculations indicate that the best water quality was in 2020, primarily influenced by the lowest recorded concentrations of turbidity parameters and total phosphate compared to other years. Conversely, the worst water quality was in 2019, predominantly due to high fecal coliform parameters, which significantly exceeded the concentrations found in other years.

The highest water quality, according to the PI method, was achieved in 2022. The TDS parameter heavily influenced this result, bearing the third-lowest value after those recorded in 2013 and 2016. Furthermore, the dissolved oxygen (DO) parameter, meeting quality standards and only exceeding 2013's concentration, also contributed.

**Fig. 8:**
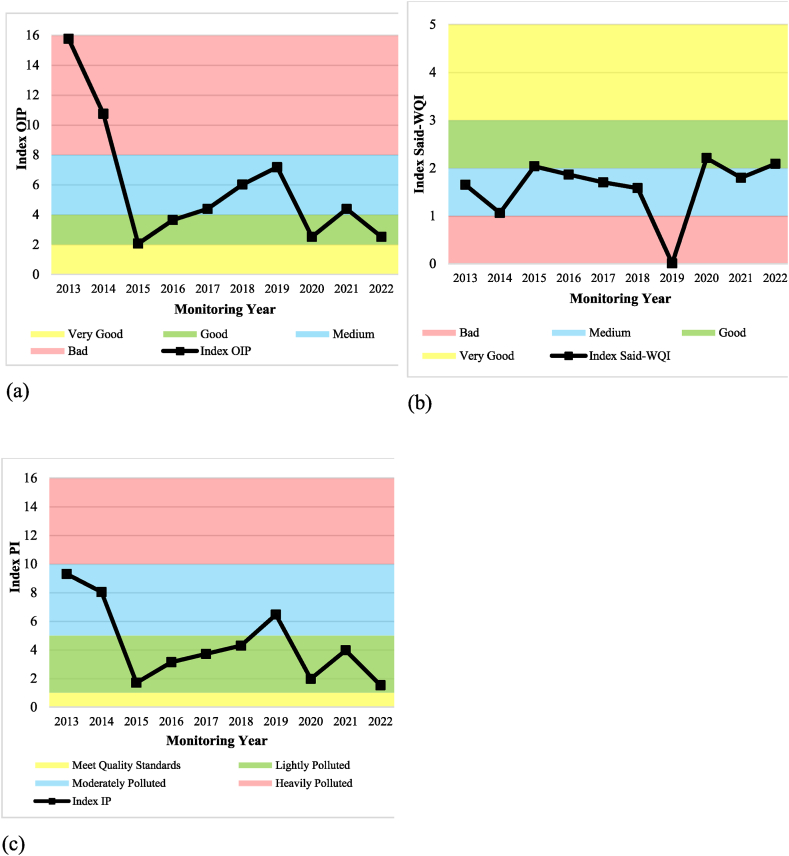
WQI graph for secondary data per year monitoring using (a) OIP, (b) Said-WQI, and (c) PI method.

Conversely, the poorest water quality was observed in 2013. The biochemical oxygen demand, nitrate, and total coliform parameters significantly influenced this, exhibiting the highest values compared to other monitored years. The total phosphate parameter, with the second-highest value after 2015, also played a role. Fig. 8a shows the graph of the OIP method calculation results, Fig. 8b shows the graph of the Said-WQI calculation results, and Fig. 8c shows the IP method results.

The results of the calculations reveal a significant improvement in water quality in 2015, attributed to the initiation of the *Citarum Bestari* Program in 2013. This program, designed to create a Clean, Healthy, Beautiful, and Sustainable environment, was set to run until 2018. However, the water quality declined between 2016 and 2018 due to the program's implementation inefficiencies. In response to this, a new initiative, *Citarum Harum*, was launched in 2018. This program was formally established under Presidential Regulation Number 15 of 2018, aiming to accelerate pollution control and restore the Citarum River Basin [36]. This initiative is planned to continue until 2025.

#### 3.4.3 Based on wet month – dry month

The results of the index calculations consistently indicate better water quality during wet months than in dry months across all monitoring points. This pattern is due to the lower values of several parameters during wet months, including electrical conductivity, TDS, turbidity, total phosphate, biochemical oxygen demand, pH, and fecal coliform concentrations.

The OIP method revealed optimal water quality results during the wet month at the Wangisagara monitoring point (Point 1). These findings are largely attributed to the lowest values of TDS, BOD, and total coliform parameters, as well as the highest DO parameters observed in comparison to other wet-dry months. Conversely, the worst water quality was detected during the dry month at the Nanjung monitoring point (Point 4). This is predominantly due to the highest TDS, BOD, and total coliform parameters, coupled with the lowest DO parameters, when compared to other wet-dry months.

The Said-WQI method's calculations suggest that the highest water quality is present during the wet month at monitoring point 1, Wangisagara. This high quality is primarily due to the second lowest values of turbidity, total phosphate, and fecal coliform in comparison to data from other wet-dry months. Conversely, the lowest water quality was noted during the dry month at monitoring point 4, Nanjung. This poor quality is significantly influenced by the highest total phosphate and the lowest dissolved oxygen (DO) parameters relative to other wet-dry month data.

**Fig. 9:**
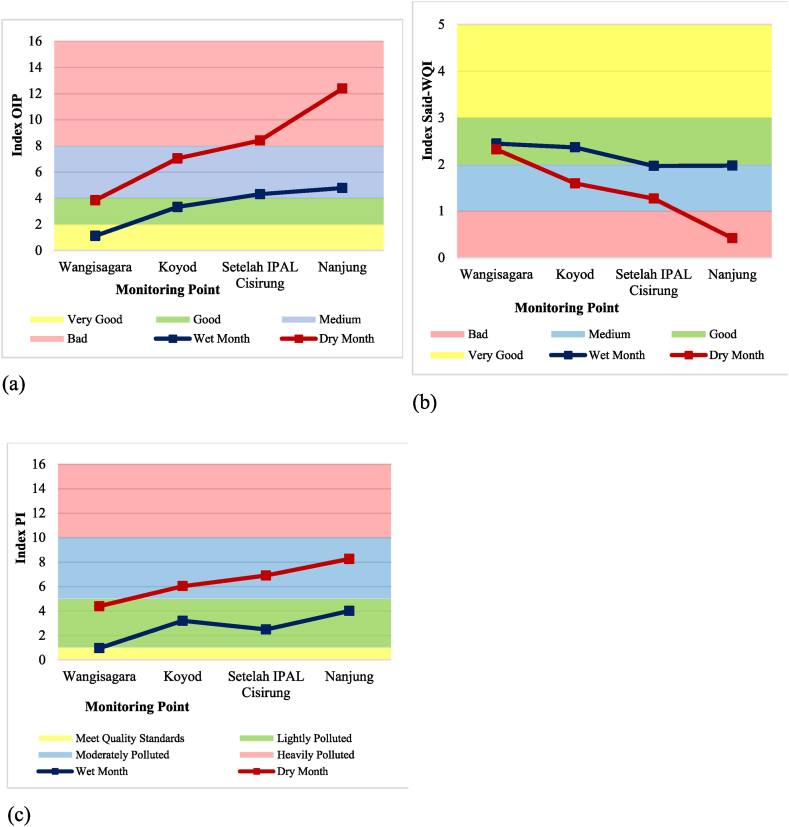
WQI graph for wet and dry month secondary data using (a) OIP, (b) Said-WQI, and (c) PI method.

Similar to the previous two methods, water quality results from the PI method were observed during the wet season at monitoring point 1 (Wangisagara). These results were heavily influenced by the lower levels of TDS, BOD, and total coliform parameters, as well as the higher levels of DO parameters, compared to data from other periods of fluctuating wet and dry months. Point 4 (Nanjung) showed the poorest water quality. It was significantly affected by high levels of TDS, total phosphate, BOD, and total coliform parameters, as well as low DO levels, compared to the parameters observed across other wet-dry month data. Table 8 recapitulates the indices and classifications of the three methods, while Fig. 9a presents the calculation results for the OIP method, Fig. 9b presents the calculation results for the Said-WQI method, and Fig. 9c presents the calculation results for the IP method.

**Table 8:** Upper Citarum River classification for wet-dry month secondary data.

Monitoring Points		Water Quality Index Calculation Method					
		OIP		Said-WQI		PI	
		Index	Classification	Index	Classification	Index	Classification
1	Wet	1.11	Very Good	2.45	Good	0.95	Meet Quality Standards
	Dry	3.83	Good	2.32	Good	4.38	Lightly Polluted
2	Wet	3.32	Good	2.36	Good	3.18	Lightly Polluted
	Dry	7.03	Medium	1.59	Medium	6.01	Moderately Polluted
3	Wet	4.30	Medium	1.97	Medium	2.47	Lightly Polluted
	Dry	8.39	Bad	1.27	Medium	6.89	Moderately Polluted
4	Wet	4.76	Medium	1.97	Medium	3.99	Lightly Polluted
	Dry	12.37	Bad	0.42	Bad	8.25	Moderately Polluted

Dissolved oxygen (DO) values are often higher during rainy months due to the reaeration process, where oxygen from the air is absorbed into the water, facilitated by increased rainfall. Conversely, DO concentrations tend to drop in drier months as water temperatures rise. When considering other parameters, concentrations tend to be lower in rainy months compared to dry months, largely attributed to dilution factors. Heavy rainfall during wet months can benefit the overall quality of water bodies. The heightened water discharge during these periods assists in the dilution and self-purification processes of rivers [37].

#### 3.4.4 Water quality based on wet and dry years

The OIP and PI methods yield superior water quality results during dry years, as opposed to wet years, at every monitoring point. This outcome is influenced by the lower concentrations of turbidity, total phosphate, nitrate, and total coliform parameters during dry seasons. On the other hand, the Said-WQI method produced contrasting results, showing better water quality during wet years, particularly at monitoring points 2, 3, and 4. Table 9 summarizes the indices and classifications yielded by the three methods.

**Table 9:** Upper Citarum River classification for secondary data for the wet-dry year.

Monitoring Points		Water Quality Index Calculation Method					
		OIP		Said-WQI		PI	
		Index	Classification	Index	Classification	Index	Classification
1	Wet	4.20	Medium	2.21	Good	5.07	Moderately Polluted
	Dry	1.28	Very Good	2.53	Good	1.05	Lightly Polluted
2	Wet	7.34	Medium	1.81	Medium	6.64	Moderately Polluted
	Dry	5.61	Medium	1.62	Medium	3.31	Lightly Polluted
3	Wet	8.70	Bad	1.51	Medium	7.40	Moderately Polluted
	Dry	6.68	Medium	1.21	Medium	4.99	Lightly Polluted
4	Wet	14.32	Bad	1.08	Medium	8.88	Moderately Polluted
	Dry	7.39	Medium	0.18	Bad	5.63	Moderately Polluted

**Fig. 10:**
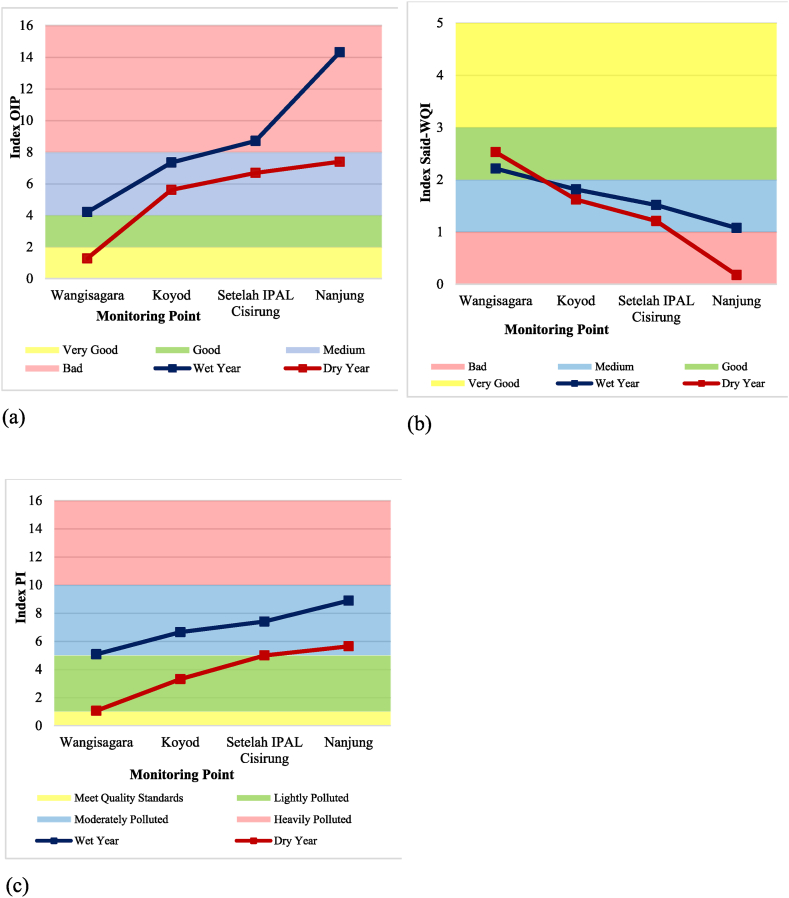
WQI graph for wet and dry year secondary data using (a) OIP, (b) Said-WQI, and (c) PI method.

The differences in water quality classification outcomes among various methods stem from the disparity in the parameters utilized. The OIP and PI methods demonstrate superior water quality results at monitoring point 1. These superior results are significantly impacted by parameters such as TDS, turbidity, biochemical oxygen demand (BOD), nitrate, and total coliform, which are at their lowest values. Furthermore, the dissolved oxygen (DO) parameters are at their highest values. Monitoring point 4 displays the poorest water quality, significantly impacted by high values of turbidity and total coliform parameters, as compared to the same parameters in other recent wet-year data. The results of these methods are illustrated in Fig. 10a for the OIP method, Fig. 10b for Said-WQI, and Fig. 10c for the IP method.

The variation in water quality outcomes among the three methods is affected by the individualized usage of parameters for every method. The Said-WQI method incorporates parameters like electrical conductivity and fecal coliform, which exhibit lower values during dry years than wet years. The reduction of microorganisms during dry years due to less water discharge leads to a decrease in waste flowing to the sea [38]. This results in lower coliform values as the microorganisms that influence this are present in smaller numbers in water bodies [35]. Similarly, the dissolved oxygen (DO) parameter shows higher values in dry years than in wet years.

### 3.5 Comparison of the use of the WQI method

The different Water Quality Index (WQI) calculation methods employed in this study each have distinctive features, extending from the calculation stages to the final results. Each method comes with its own **advantages and limitations**, which should be carefully considered when selecting an index calculation method going forward. Table 10 illustrates the differences among the three methods under consideration in this study.

**Table 10:** Comparison of index calculation method.

Indicator	OIP method	Said-WQI methods	PI methods
Origin of method (Country)	India	America	American (but has been commonly used in Indonesia)
Use of Methods	Can be used as a scheme Surface water quality classification **Advantage:** Can be used as a tool for formulating pollution control strategies **Limitation:** Cannot represent specific water conditions. It cannot be used to determine the presence of metal contamination in rivers.	Can represent water quality in general **Limitation:** It cannot be used to represent specific water conditions. It cannot be used to determine the presence of metal contamination in rivers.	Used to determine the degree of pollution relative to parameters **Advantage:** Applicable all parts of water bodies
Types of Parameter Selection	*Mixed System*	*Fixed System*	*Open System*
Parameter Selection Method	Based on water quality standards applicable in India	Based on expert opinion	No specific parameters required
Parameters used	13 Parameters are used, but the number of parameters can be adjusted to the needs of the user. **Parameter:** Turbidity, pH, color, DO, BOD, TDS, hardness, Cl, SO_4_, NO_3_, Total coliform, As and F	5 parameters are used. The number of parameters cannot be changed **Parameter:** Electrical conductivity, total phosphate, DO, turbidity, and fecal coliform	The number of parameters is not specified and can be adjusted according to needs
Sub-index transformations	Mathematical equations and different function curves for each parameter	No sub-index transforms were performed	Mathematical equations, especially for certain parameters
Weighting	Equivalent	No weighting	No weighting
Aggregation process	Average of sub-indices	mathematical equations	Equations
Classification	OIP Scale: • 0–2: Very Good • 2–4: Good • 4–8: Medium • 8–16: Bad	Said-WQI Scale: • 0–1: Bad • 1–2: Medium • 2–3: Good • Very Good	PI Scale: • 0 ≤ PIj ≤1: Meet quality standards (good condition) • 1 < PIj ≤5: Lightly polluted • 5 < PIj ≤10: Moderately Polluted • PIj >10: Heavily Polluted

### 3.6 Analysis of index calculation results using correlation test

The correlation test was conducted to compare the closeness of the relationship between the index calculation results using other methods and the index calculation method in Indonesia. This correlation test was calculated using Microsoft Excel software, evaluated the degree of correlation between methods across various monitoring conditions, including monitoring points, monitoring year, wet and dry months, wet and dry years, as well as primary and secondary data.

#### 3.6.1 Monitoring points

Correlation tests were conducted to evaluate the water quality index results at monitoring points for primary and secondary data. Table 11 shows the correlation results between WQI methods based on primary data monitoring, while Table 12 shows the correlation results between WQI methods and secondary data monitoring.

**Table 11:** Correlation between WQI methods per monitoring point (primary data).

*Correlation between*	*OIP*	*Said-WQI*	*PI*
OIP	1		
Said-WQI	−0.9823	1	
PI	0.9495	−0.9160	1

**Table 12:** Correlation between WQI methods per monitoring point (secondary data).

*Correlation between*	*OIP*	*Said-WQI*	*PI*
OIP	1		
Said-WQI	−0.9990	1	
PI	0.9932	−0.9955	1

Based on the correlation test results of the WQI method on the primary data of the monitoring points, the correlation value between the PI method and the OIP method is 0.9495, which shows a strong positive correlation relationship. The relationship between the method developed by Said-WQI and OIP shows a negative correlation, indicating the method developed by Said-WQI has a weak relationship with the PI method. Similar trends were observed in the correlation test results at the monitoring point for secondary data. The PI method has a perfect relationship with the OIP method, with a correlation value of 0.9932, while a negative correlation persisted with the Said-WQI developed method.

#### 3.6.2 Annual monitoring

The correlation test between the PI method and the OIP method's water quality index showed a perfect positive correlation, with a value of 0.9642. Based on the correlation test results, the correlation between the PI method and the WQI method developed by Said, shows a moderate but negative correlation, with a value of −0.6104. Table 13 below shows the correlation test results for the OIP method, PI, and the method developed by Said using secondary data monitored annually.

**Table 13:** Correlation of each WQI method on annual monitoring data (secondary data).

*Correlation between*	*OIP*	*Said-WQI*	*PI*
OIP	1		
Said-WQI	−0.4195	1	
PI	0.9642	−0.6104	1

#### 3.6.3 Wet month and dry month data

The water quality index was calculated based on wet and dry months using both primary and secondary data sources. The results of the water quality index in the wet month - dry month group were then tested for correlation based on the type of primary data and the type of secondary data.

Based on Table 14, the correlation test for primary data shows a perfect correlation between the PI and OIP methods, with a correlation result of 0.9169. However, the correlation test between the PI method and the WQI method developed by Said showed negative results, which can be interpreted as having a weak correlation. Similar findings were observed in the secondary data, where the PI method and the OIP method have a perfect correlation with a correlation value of 0.9499, as shown in Table 15.

**Table 14:** Correlation results between WQI method in wet month - dry month group (primary data).

*Correlation between*	*OIP*	*Said-WQI*	*PI*
OIP	1		
Said-WQI	−0.9804	1	
PI	0.9169	−0.8764	1

**Table 15:** Correlation results between WQI method in wet month - dry month group (secondary data).

*Correlation between*	*OIP*	*Said-WQI*	*PI*
OIP	1		
Said-WQI	−0.9833	1	
PI	0.9499	−0.8885	1

#### 3.6.4 Wet-year and dry-year data

The correlation results between WQI methods in wet years - dry years show that the PI method has a perfect correlation with the OIP method. The perfect correlation result is shown with a value of 0.9094. The weak correlation between the PI method and the Said-WQI method is indicated by a value of −0.5037, as shown in Table 16.

**Table 16:** Correlation between WQI methods based on wet year - dry year (secondary data).

	OIP	Said-WQI	PI
OIP	1		
Said-WQI	−0.5973	1	
PI	0.9094	−0.5037	1

#### 3.6.5 Comparison of primary data and secondary data

In this study, a correlation test was conducted to compare primary data and secondary data for each method. The results showed a **perfect correlation** between the primary and secondary data across all methods. This means that the water quality index calculated based on primary data and secondary data does not have a significant difference. The correlation test results for primary and secondary data using the WQI OIP method are shown in Table 17, for the Said-WQI method are shown in Table 18, and for the PI method are shown in Table 19.

**Table 17:** Correlation of primary data and secondary data of OIP method.

	OIP Primary data	OIP Secondary data
OIP Primary data	1	
OIP Secondary data	0.9219	1

**Table 18:** Correlation of primary data and secondary data WQI method developed by Said.

	Said-WQI Primary data	Said-WQI Secondary data
Said-WQI Primary data	1	
Said-WQI Secondary data	0.9642	1

**Table 19:** Correlation of primary data and secondary data of PI method.

	PI Primary data	PI Secondary data
PI Primary data	1	
PI Secondary data	0.8705	1

## 4 Conclusion

The assessment of water quality in the Upper Citarum River, conducted using the OIP, Said-WQI, and PI methods, revealed inconsistencies among the three methods. These differences largely stem from varying concentration values of the employed parameters. According to OIP and Said-WQI calculation results, the river's status varies from ‘Good’ to ‘Moderate’ and ‘Poor’. In contrast, the PI method classified the river as ‘Mildly Polluted’, ‘Moderately Polluted’, and ‘Severely Polluted’. An analysis of the Upper Citarum River's secondary monitoring data showed that parameters such as TDS, total phosphate, pH, and nitrate meet the class 2 quality standards outlined in Appendix VI of the Government Regulation Number 22 of 2021. However, BOD, DO, fecal coliform, and total coliform parameters did not meet the same standards. A similar analysis using primary data indicated that while the TDS, total phosphate, DO, pH, and nitrate parameters meet the quality standards, BOD, fecal coliform, and total coliform parameters failed to comply with the same standards.

Based on the research conducted, there is potential for further refinement and development of each index calculation method. Further studies can be conducted to expand the range of parameters assessed, especially for the OIP and IP methods, to provide a more comprehensive understanding of water quality. Additionally, future studies could broaden the scope of the study to include all monitoring points across the entire length from upstream to downstream of the Citarum River.

## CRediT authorship contribution statement

**Mariana Marselina:** Writing – review & editing, Supervision, Resources, Methodology, Conceptualization. **Nurul Aulia Rahmi:** Writing – original draft, Methodology, Data curation. **Siti Ai Nurhayati:** Writing – review & editing, Visualization, Validation.

## Data availability statement

All relevant data are included in the paper or its Supplementary Information.

## Funding

The author(s) disclosed receipt of the following financial support for the research, authorship, and/or publication of this article: This research was funded by 10.13039/501100015689ITB Research Grant Year of 2024.

## Declaration of competing interest

The authors declare that they have no known competing financial interests or personal relationships that could have appeared to influence the work reported in this paper.
