# Supplementary material for: The water quality of the Upper Citarum: Applying the overall index of pollution, Said-WQI, and pollution index methods
Source: Heliyon. 2025 Jan 8;11(2):e41690. doi: 10.1016/j.heliyon.2025.e41690 (PMC11787455; doi:10.1016/j.heliyon.2025.e41690)
Supplement: Multimedia component 1 [file mmc1.docx]

**S A L I N A N**

KEPUTUSAN

MENTERI NEGARA LINGKUNGAN HIDUP NOMOR : 115 TAHUN 2003

TENTANG

PEDOMAN PENENTUAN STATUS MUTU AIR MENTERI NEGARA LINGKUNGAN HIDUP,

Menimbang : bahwa untuk melaksanakan ketentuan Pasal 14 ayat (2) Peraturan Pemerintah Nomor 82 Tahun 2001 tentang Pengelolaan Kualitas Air dan Pengendalian Pencemaran Air maka dipandang perlu menetapkan Keputusan Menteri Negara Lingkungan Hidup tentang Pedoman Penentuan Status Mutu Air;

Mengingat : 1. Undang-undang Nomor 23 Tahun 1997 tentang Pengelolaan Lingkungan Hidup (Lembaran Negara Tahun 1997 Nomor 68, Tambahan Lembaran Negara Nomor 3699);

1. Undang-undang Nomor 22 Tahun 1999 tentang Pemerintahan Daerah (Lembaran Negara Tahun 1999 Nomor 60, Tambahan Lembaran Negara Nomor 3839);
2. Peraturan Pemerintah Nomor 27 Tahun 1999 tentang Analisis Mengenai Dampak Lingkungan Hidup (Lembaran Negara Tahun 1999 Nomor 59, Tambahan Lembaran Negara Nomor 3838);
3. Peraturan Pemerintah Nomor 25 Tahun 2000 tentang Kewenangan Pemerintah dan Kewenangan Provinsi Sebagai Daerah Otonom (Lembaran Negara Tahun 2000 Nomor 54, Tambahan Lembaran Negara Nomor 3952);
4. Peraturan Pemerintah Nomor 82 Tahun 2001 tentang Pengelolaan Kualitas Air dan Pengendalian Pencemaran Air (Lembaran Negara Tahun 2001 Nomor 153, Tambahan Lembaran Negara Nomor 4161);
5. Keputusan Presiden Republik Indonesia Nomor 2 Tahun 2002 tentang Perubahan Atas Keputusan Presiden Nomor 101 Tahun 2001 tentang Kedudukan, Tugas, Fungsi, Kewenangan, Susunan Organisasi, Dan Tata Kerja Menteri Negara;

M E M U T U S K A N :

Menetapkan : KEPUTUSAN MENTERI NEGARA LINGKUNGAN HIDUP TENTANG PEDOMAN PENENTUAN STATUS MUTU AIR.

Pasal 1 Dalam keputusan ini yang dimaksud dengan :

1. Mutu air adalah kondisi kualitas air yang diukur dan atau diuji

berdasarkan parameter-parameter tertentu dan metode tertentu berdasarkan peraturan perundang-undangan yang berlaku.

1. Status mutu air adalah tingkat kondisi mutu air yang menunjukkan kondisi cemar atau kondisi baik pada suatu sumber air dalam waktu tertentu dengan membandingkan dengan baku mutu air yang ditetapkan.
2. Sumber air adalah wadah air yang terdapat di atas dan di bawah permukaan tanah, termasuk dalam pengertian ini akuifer, mata air, sungai, rawa, danau, situ, waduk, dan muara.

Pasal 2

1. Penentuan status mutu air dapat menggunakan Metoda STORET atau Metoda Indeks Pencemaran.
2. Pedoman untuk menentukan status mutu air dengan Metoda STORET dilakukan sesuai dengan pedoman pada Lampiran I Keputusan ini.
3. Pedoman untuk menentukan status mutu air dengan Metoda Indeks Pencemaran dilakukan sesuai dengan pedoman pada Lampiran II Keputusan ini.

Pasal 3

1. Apabila timbul kebutuhan untuk menggunakan metoda lain yang juga berdasarkan kaidah ilmu pengetahuan dan teknologi untuk menyesuaikan dengan situasi dan kondisi serta kapasitas daerah, maka

dapat digunakan metoda di luar metoda sebagaimana dimaksud dalam Pasal 2.

1. Metoda sebagaimana dimaksud dalam ayat (1) digunakan setelah mendapat rekomendasi dari instansi yang bertanggung jawab di bidang pengelolaan lingkungan hidup dan pengendalian dampak lingkungan.

Pasal 4

Dalam jangka waktu selambat-lambatnya 1 (satu) tahun sejak ditetapkan Keputusan ini, status mutu air yang telah ditetapkan sebelumnya wajib disesuaikan dengan ketentuan dalam Keputusan ini.

Pasal 5

Pada saat berlakunya Keputusan ini semua peraturan perundang-undangan yang berkaitan dengan status mutu air yang telah ada tetap berlaku sepanjang tidak bertentangan dengan Keputusan ini.

Pasal 6

Keputusan ini mulai berlaku pada tanggal ditetapkan.

Ditetapkan di : Jakarta pada tanggal : 10 Juli 2003

Menteri Negara Lingkungan Hidup,

**ttd**

**Nabiel Makarim, MPA, MSM.**

**Salinan sesuai dengan aslinya**

**Deputi MENLH Bidang Kebijakan dan Kelembagaan Lingkungan Hidup,**

**Hoetomo, MPA**

Lampiran I

Keputusan Menteri Negara Lingkungan Hidup.

Nomor : 115 Tahun 2003

Tanggal: 10 Juli 2003

PENENTUAN STATUS MUTU AIR DENGAN METODA STORET

1. Uraian Metoda STORET

Metoda STORET merupakan salah satu metoda untuk menentukan status mutu air yang umum digunakan. Dengan metoda STORET ini dapat diketahui parameter-parameter yang telah memenuhi atau melampaui baku mutu air.

Secara prinsip metoda STORET adalah membandingkan antara data kualitas air dengan baku mutu air yang disesuaikan dengan peruntukannya guna menentukan status mutu air.

Cara untuk menentukan status mutu air adalah dengan menggunakan sistem nilai dari “US-EPA (*Environmental Protection Agency*)” dengan mengklasifikasikan mutu air dalam empat kelas, yaitu :

- 1. Kelas A : baik sekali, skor = 0 🡪 memenuhi baku mutu
  2. Kelas B : baik, skor = -1 s/d -10 🡪 cemar ringan
  3. Kelas C : sedang, skor = -11 s/d -30 🡪 cemar sedang
  4. Kelas D : buruk, skor ≥ -31 🡪 cemar berat

1. Prosedur Penggunaan

Penentuan status mutu air dengan menggunakan metoda STORET dilakukan dengan langkah-langkah sebagai berikut :

1. Lakukan pengumpulan data kualitas air dan debit air secara periodik sehingga membentuk data dari waktu ke waktu (*time series data*).
2. Bandingkan data hasil pengukuran dari masing-masing parameter air

dengan nilai baku mutu yang sesuai dengan kelas air.

1. Jika hasil pengukuran memenuhi nilai baku mutu air (hasil pengukuran < baku mutu) maka diberi skor 0.
2. Jika hasil pengukuran tidak memenuhi nilai baku mutu air (hasil pengukuran > baku mutu), maka diberi skor :

Tabel 1.1. Penentuan sistem nilai untuk menentukan status mutu air

| Jumlah contoh1) | Nilai | Parameter | | |
| --- | --- | --- | --- | --- |
|  |  | Fisika | Kimia | Biologi |
| < 10 | Maksimum | -1 | -2 | -3 |
|  | Minimum | -1 | -2 | -3 |
|  | Rata-rata | -3 | -6 | -9 |
| ≥ 10 | Maksimum | -2 | -4 | -6 |
|  | Minimum | -2 | -4 | -6 |
|  | Rata-rata | -6 | -12 | -18 |

Sumber : Canter (1977)

Catatan : 1) jumlah parameter yang digunakan untuk penentuan status mutu air.

1. Jumlah negatif dari seluruh parameter dihitung dan ditentukan status mutunya dari jumlah skor yang didapat dengan menggunakan sistem nilai.
2. Contoh Perhitungan

Untuk lebih jelasnya, dapat dilihat pada contoh berikut ini. Tabel 1.2. merupakan contoh penerapan penentuan kualitas air menurut metoda STORET yang dilakukan oleh Unpad, Bandung. Data diambil dari sungai Ciliwung pada stasiun 1. Pada tabel ini tidak diberikan data lengkap hasil analisa di sungai Ciliwung, tetapi hanya diberikan nilai maksimum, minimum, dan rata-rata dari data-data hasil.

Cara pemberian skor untuk tiap parameter adalah sebagai berikut (contoh, untuk Hg):

1. Hg merupakan parameter kimia, maka gunakan skor untuk parameter kimia.
2. Kadar Hg yang diharapkan untuk air golongan C adalah 0.002 mg/l.
3. Kadar Hg maksimum hasil pengukuran adalah 0.0296 mg/l, ini berarti kadar Hg melebihi baku mutunya. Maka skor untuk nilai maksimum adalah -2.
4. Kadar Hg minimum hasil pengukuran adalah 0.0006 mg/l, ini berarti kadar Hg sesuai dengan baku mutunya. Maka skornya adalah 0.
5. Kadar Hg rata-rata hasil pengukuran adalah 0.0082 mg/l, ini berarti melebihi baku mutunya. Maka skornya adalah –6.
6. Jumlahkan skor untuk nilai maksimum, minimum, dan rata-rata. Untuk Hg pada contoh ini skor Hg adalah –8.
7. Lakukan hal yang sama untuk tiap parameter, apabila tidak ada baku mutunya untuk parameter tertentu, maka tidak perlu dilakukan perhitungan.
8. Jumlahkan semua skor, ini menunjukan status mutu air. Pada contoh ini skor total adalah –58, ini berarti sungai Ciliwung pada stasiun 1 mempunyai mutu yang buruk untuk peruntukan golongan C.

Tabel 1.2. Status Mutu Kualitas Air Menurut Sistem Nilai STORET

di Stasiun 1 sungai Ciliwung bagi peruntukan Golongan C (PP 20/1990)

| No. | Parameter | Satuan | Baku  Mutu | Hasil Pengukuran | | | Skor |
| --- | --- | --- | --- | --- | --- | --- | --- |
|  |  |  |  | Maksimu  m | Minimum | Rata-rata |  |
|  | FISIKA |  |  |  |  |  |  |
| 1 | TDS | mg/l |  | 289 | 179,4 | 224,2 |  |
| 2 | Suhu air | C | normal +  3 | 24,15 | 20,5 | 22,06 | 0 |
| 3 | DHL | < mhos/c  m |  | 82,6 | 72 | 76,3 |  |
| 4 | Kecerahan | M |  | 0,46 | 0,35 | 0,41 |  |
|  | KIMIA |  |  |  |  |  |  |
|  | a. Anorganik |  |  |  |  |  |  |
| 1 | Hg | mg/l | 0,002 | 0,0296 | 0,0006 | 0,0082 | -8 |
| 2 | As | mg/l | 0,5 | 0,0014 | Tt | 0,0004 | 0 |
| 3 | Ba | mg/l | 1,5 | 17,401 | 11,239 | 15,3665 |  |
| 4 | F | mg/l | 0,01 | 0,51 | 0,28 | 0,4138 | 0 |

| 5 | Cd | mg/l | nihil | Tt | Tt | Tt | 0 |
| --- | --- | --- | --- | --- | --- | --- | --- |
| 6 | Cr (VI) | mg/l |  | 0,0036 | Tt | 0,0009 | -8 |
| 7 | Mn | mg/l |  | 0,033 | Tt | 0,083 |  |
| 8 | Na | mg/l |  | 15,421 | 5,1672 | 11,0246 |  |
| 9 | NO3-N | mg/l |  | 12,28 | 0,04 | 3,4675 |  |
| 10 | NO2-N | mg/l | 0,06 | 1 | 0,0075 | 0,3996 | -8 |
| 11 | NH3-N | mg/l | 0,02 | 1,53 | Tt | 0,576 | -8 |
| 12 | pH |  | 6-8.5 | 7,83 | 6,72 | 7,41 | 0 |
| 13 | Se | mg/l | 0,05 | Tt | Tt | Tt | 0 |
| 14 | Zn | mg/l | 0,02 | 0,0457 | Tt | 0,0114 | -2 |
| 15 | CN | mg/l | 0,01 | Tt | Tt | Tt | 0 |
| 16 | SO4 | mg/l |  | 40 | 2,2 | 14,175 |  |
| 17 | H2S | mg/l | 0,002 | 1,27 | 0,0014 | 0,3354 | -8 |
| 18 | Cu | mg/l | 0,02 | 0,008 | Tt | 0,0043 | 0 |
| 19 | Pb | mg/l | 0,03 | 0,2456 | Tt | 0,1451 | -8 |
| 20 | RSC | mg/l |  | 3,42 | 2,42 | 2,985 |  |
| 21 | BOD5 | mg/l |  | 42,51 | 22,97 | 32,92 |  |
| 22 | COD | mg/l |  | 62,2 | 34,32 | 48,08 |  |
| 23 | Minyak dan  lemak | mg/l | 0,5 | Tt | Tt | Tt | 0 |
| 24 | PO4 | mg/l |  | 2,28 | 0,02 | 0,7167 |  |
| 25 | Phenol | mg/l | 0,001 | Tt | Tt | Tt | 0 |
| 26 | Cl2 | mg/l | 0,003 | 1,3315 | 0,0003 | 0,3383 | -8 |
| 27 | B | mg/l |  | 2,103 | 0,81 | 1,4575 |  |
| 28 | COD | mg/l |  | 0,1242 | 0,0145 | 0,0653 |  |
| 29 | Ni | mg/l |  | Tt | Tt | Tt |  |
| 30 | HCO3 | mg/l |  | - | - | - |  |
| 31 | CO2-bebas | mg/l |  | 11,88 | 7,92 | 9,24 |  |
| 32 | Salinitas | 0/00 |  | 0,02 | 0 | 0,015 |  |
| 33 | DO | mg/l | > 3 | 9,1 | 8 | 8,433 | 0 |
|  | b. Organik |  |  |  |  |  |  |
| 1 | Aldrin | mg/l |  | Tt | Tt | Tt |  |
| 2 | Dieldrin | mg/l |  | Tt | Tt | Tt |  |
| 3 | Chlordane | mg/l |  | Tt | Tt | Tt |  |
| 4 | DDT | mg/l | 0,002 | Tt | Tt | Tt | 0 |
| 5 | Detergent | mg/l | 0,2 | Tt | Tt | Tt | 0 |
| 6 | Lindane | mg/l |  | Tt | Tt | Tt |  |
| 7 | PCB | mg/l |  | Tt | Tt | Tt |  |
| 8 | Endrine | mg/l | 0,004 | Tt | Tt | Tt | 0 |
| 9 | BHC |  | 0,21 | Tt | Tt | Tt | 0 |
|  | MIKROBIOLO  GI |  |  |  |  |  |  |
| 1 | Coliform tinja | Jml/100  ml |  | 15x10^6 | 2.5x10^6 | 7.125x10^6 |  |

| 2 | Total coliform | Jml/100  ml |  | 15x10^6 | 2.5x10^6 | 8.375x10^6 |  |
| --- | --- | --- | --- | --- | --- | --- | --- |
|  | Jumlah Skor |  |  |  |  |  | -58 |

Ditetapkan di : Jakarta pada tanggal : 10 Juli 2003

Menteri Negara Lingkungan Hidup,

**ttd**

**Nabiel Makarim, MPA, MSM**

**Salinan sesuai dengan aslinya**

**Deputi MENLH Bidang Kebijakan dan Kelmbagaan Lingkungan Hidup,**

**Hoetomo, MPA.**

Lampiran II

Keputusan Menteri Negara Lingkungan Hidup.

Nomor : 115 Tahun 2003

Tanggal: 10 Juli 2003

PENENTUAN STATUS MUTU AIR DENGAN METODA INDEKS PENCEMARAN

- 1. Uraian Metode Indeks Pencemaran

Sumitomo dan Nemerow (1970), Universitas Texas, A.S., mengusulkan suatu indeks yang berkaitan dengan senyawa pencemar yang bermakna untuk suatu peruntukan. Indeks ini dinyatakan sebagai Indeks Pencemaran *(Pollution Index*) yang digunakan untuk menentukan tingkat pencemaran relatif terhadap parameter kualitas air yang diizinkan (Nemerow, 1974). Indeks ini memiliki konsep yang berlainan dengan Indeks Kualitas Air (*Water*

*Quality Index*). Indeks Pencemaran (IP) ditentukan untuk suatu peruntukan,

kemudian dapat dikembangkan untuk beberapa peruntukan bagi seluruh bagian badan air atau sebagian dari suatu sungai.

Pengelolaan kualitas air atas dasar Indeks Pencemaran (IP) ini dapat memberi masukan pada pengambil keputusan agar dapat menilai kualitas badan air untuk suatu peruntukan serta melakukan tindakan untuk memperbaiki kualitas jika terjadi penurunan kualitas akibat kehadiran senyawa pencemar. IP mencakup berbagai kelompok parameter kualitas yang independent dan bermakna.

- 1. Definisi

Jika Lij menyatakan konsentrasi parameter kualitas air yang dicantumkan dalam Baku Peruntukan Air (j), dan Ci menyatakan konsentrasi parameter kualitas air (i) yang diperoleh dari hasil analisis cuplikan air pada suatu lokasi pengambilan cuplikan dari suatu alur sungai, maka PIj adalah Indeks Pencemaran bagi peruntukan (j) yang merupakan fungsi dari Ci/Lij.

PIj = < (C1/L1j, C2/L2j,…,Ci/Lij) (2-1)

Tiap nilai Ci/Lij menunjukkan pencemaran relatif yang diakibatkan oleh parameter kualitas air. Nisbah ini tidak mempunyai satuan. Nilai Ci/Lij = 1,0 adalah nilai yang kritik, karena nilai ini diharapkan untuk dipenuhi bagi suatu Baku Mutu Peruntukan Air. Jika Ci/Lij >1,0 untuk suatu parameter, maka konsentrasi parameter ini harus dikurangi atau disisihkan, kalau badan air digunakan untuk peruntukan (j). Jika parameter ini adalah parameter yang bermakna bagi peruntukan, maka pengolahan mutlak harus dilakukan bagi air itu.

Pada model IP digunakan berbagai parameter kualitas air, maka pada penggunaannya dibutuhkan nilai rata-rata dari keseluruhan nilai Ci/Lij sebagai tolok-ukur pencemaran, tetapi nilai ini tidak akan bermakna jika salah satu nilai Ci/Lij bernilai lebih besar dari 1. Jadi indeks ini harus mencakup nilai Ci/Lij yang maksimum

PIj = < {(Ci/Lij)R,(Ci/Lij)M} (2-2)

Dengan (Ci/Lij)R : nilai ,Ci/Lij rata-rata (Ci/Lij)M : nilai ,Ci/Lij maksimum

Jika (Ci/Lij)R merupakan ordinat dan (Ci/Lij)M merupakan absis maka PIj merupakan titik potong dari (Ci/Lij)R dan (Ci/Lij)M dalam bidang yang dibatasi oleh kedua sumbu tersebut.

(C_i_/L_ij_)_R_

PI_j_

(C_i_/L_ij_)_M_

Gambar 2.1. Pernyataan Indeks untuk suatu Peruntukan (j)

Perairan akan semakin tercemar untuk suatu peruntukan (j) jika nilai (Ci/Lij)R dan atau (Ci/Lij)M adalah lebih besar dari 1,0. Jika nilai maksimum Ci/Lij dan atau nilai rata-rata Ci/Lij makin besar, maka tingkat pencemaran suatu badan air akan makin besar pula. Jadi panjang garis dari titik asal hingga titik Pij diusulkan sebagai faktor yang memiliki makna untuk menyatakan tingkat pencemaran.

PIj = m (2-3)

(C /L )^2^ + (C /L )^2^

i ij M i ij R

Dimana m = faktor penyeimbang

Keadaan kritik digunakan untuk menghitung nilai m

PIj = 1,0 jika nilai maksimum Ci/Lij = 1,0 dan nilai rata-rata Ci/Lij = 1,0 maka 1,0 = m

(1)^2^ + (1) ^2^

m = 1/ , maka persamaan 3-3 menjadi

2

PI_j_ =

(C /L )^2^ + (C /L )^2^

i ij M i ij R

2

……………………………………………..(2-4)

Metoda ini dapat langsung menghubungkan tingkat ketercemaran dengan dapat atau tidaknya sungai dipakai untuk penggunaan tertentu dan dengan nilai parameter-parameter tertentu.

Evaluasi terhadap nilai PI adalah :

0 ≤ PIj ≤ 1,0 🡪 memenuhi baku mutu (kondisi baik) 1,0 < PIj ≤ 5,0 🡪 cemar ringan

5,0 < PIj ≤ 10 🡪 cemar sedang PIj > 10 🡪 cemar berat

- 1. Prosedur Penggunaan

Jika Lij menyatakan konsentrasi parameter kualitas air yang dicantumkan dalam Baku Mutu suatu Peruntukan Air (j), dan Ci menyatakan konsentrasi parameter kualitas air (i) yang diperoleh dari hasil analisis cuplikan air pada suatu lokasi pengambilan cuplikan dari suatu alur sungai, maka PIj adalah

Indeks Pencemaran bagi peruntukan (j) yang merupakan fungsi dari Ci/Lij. Harga Pij ini dapat ditentukan dengan cara :

- - 1. Pilih parameter-parameter yang jika harga parameter rendah maka kualitas air akan membaik.
    2. Pilih konsentrasi parameter baku mutu yang tidak memiliki rentang.
    3. Hitung harga Ci/Lij untuk tiap parameter pada setiap lokasi pengambilan cuplikan.
    4. a. Jika nilai konsentrasi parameter yang menurun menyatakan tingkat pencemaran meningkat, misal DO. Tentukan nilai teoritik atau nilai maksimum Cim (misal untuk DO, maka Cim merupakan nilai DO jenuh). Dalam kasus ini nilai Ci/Lij hasil pengukuran digantikan oleh nilai Ci/Lij hasil perhitungan, yaitu :

(Ci/Lij)baru =

Cim - Ci (hasil pengukuran)

Cim - Lij

- - - 1. Jika nilai baku Lij memiliki rentang

- untuk Ci < Lij rata-rata

[ C - (L ) ]

(C /L ) = i ij rata-rata

^i^ ^ij^ ^baru^ { (L ) - (L ) }

ij minimum ij rata-rata

- untuk Ci > Lij rata-rata

(C /L ) =

[ Ci - (Lij )rata-rata ]

^i^ ^ij^ ^baru^ { (L ) - (L ) }

ij maksimum ij rata-rata

- - - 1. Keraguan timbul jika dua nilai (Ci/Lij) berdekatan dengan nilai acuan 1,0, misal C1/L1j = 0,9 dan C2/L2j = 1,1 atau perbedaan yang sangat besar, misal C3/L3j = 5,0 dan C4/L4j = 10,0. Dalam contoh ini tingkat kerusakan badan air sulit ditentukan. Cara untuk mengatasi kesulitan ini adalah :
         1. Penggunaan nilai (Ci/Lij)hasil pengukuran kalau nilai ini lebih kecil dari 1,0.
         2. Penggunaan nilai (Ci/Lij)baru jika nilai (Ci/Lij)hasil pengukuran lebih besar dari 1,0.

(Ci/Lij)baru = 1,0 + P.log(Ci/Lij)hasil pengukuran

P adalah konstanta dan nilainya ditentukan dengan bebas dan disesuaikan dengan hasil pengamatan lingkungan dan atau persyaratan yang dikehendaki untuk suatu peruntukan (biasanya digunakan nilai 5).

1. Tentukan nilai rata-rata dan nilai maksimum dari keseluruhan Ci/Lij ((Ci/Lij)R dan (Ci/Lij)M).
2. Tentukan harga PIj PIj =

(C /L )^2^ + (C /L )^2^

i ij M i ij R

2

- 1. Contoh Perhitungan

Pada contoh berikut ini diberikan data untuk suatu sampel sungai yang akan ditentukan indeks pencemarannya (IP). Hasil pengukuran sampel diberikan pada kolom 2 (Ci) dan baku mutu perairan tersebut diberikan pada kolom 3 (LiX). Pada contoh perhitungan hanya digunakan 6 parameter saja. Contoh yang diberikan berikut ini hanya bertujuan agar pemakai metoda Indeks Pencemaran dapat memahami cara menghitung harga PIj.

Tabel 2.2. Contoh penentuan IP untuk baku mutu x

| Parameter | Ci | LiX | Ci/LiX | Ci/LiX baru |
| --- | --- | --- | --- | --- |
| TSS | 100 | 50 | 2 | 2,5 |
| DO | 2 | 6 | 0,28 | 0,28 |
| pH | 8 | 6-9 | 0,5 | 0,5 |
| Fecal coliform | 2000 | 1000 | 2 | 2,5 |
| BOD | 8 | 2 | 4,0 | 4,0 |
| Se | 0,07 | 0,01 | 7,0 | 5,2 |

- Contoh perhitungan TSS :

C1/L1X = 100 / 50 = 2

C1/L1X > 1

Maka gunakan persamaan (Ci/Lij)baru (C1/L1X)baru = 1,0 + 5 log 2 = 2,5

Catatan : Ci/Lij baru dihitung karena nilai Ci/Lij yang berjauhan

untuk Ci/Lij < 1 digunakan Ci/Lij hasil pengukuran, tetapi bila Ci/Lij > 1 perlu dicari Ci/Lij baru.

- Contoh perhitungan DO :

DO merupakan parameter yang jika harga parameter rendah maka kualitas akan menrun. Maka sebelum menghitung C2/L2X harus dicari terlebih dahulu harga C2 baru.

DOmaks = 7 pada temperatur 250C

C2 baru = 7 – 2 = 5

7 – 6 3

C2/L2X = (5/3) / 6 = 0,28

- Contoh perhitungan pH :

Karena harga baku mutu pH memiliki rentang, maka penetuan C3/L3X dilakukan dengan cara :

L3X rata-rata = 6 + 9 = 7,5
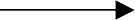
 C3 > L3X rata-rata 2

C3/L3X = ( 8 – 7,5 ) = 0,5

( 9 – 8 )

- - Tentukan nilai (Ci/LiX)R = 2,58 (nilai rata-rata dari kolom 5)
  - Tentukan nilai (Ci/LiX)M = 5,2 (nilai maksimum dari kolom 5)
  - Dengan menggunakan persamaan pada langkah no 5 (lihat prosedur 3.2), maka dapat ditentukan nilai PIX = 4,10.

Apabila kemudian data air sungai yang sama ingin dibandingkan terhadap baku mutu yang berbeda, misalnya Y (kolom II, Tabel 3.3), maka perhitungannya menjadi sebagai berikut:

Tabel 2.3. Contoh penentuan IP untuk baku mutu Y

| Parameter | Ci | LiY | Ci/LiY | Ci/LiY baru |
| --- | --- | --- | --- | --- |
| TSS | 100 | 400 | 0,25 | 0,25 |
| DO | 2 | 1 | 2 | 0,83 |
| pH | 8 | 6-9 | 0,5 | 0,5 |
| BOD | 8 | 10 | 0,8 | 0,8 |
| Se | 0,07 | 0,08 | 0,88 | 0,88 |

Dari Tabel 2.3., maka dapat ditentukan nilai-nilai berikut:

- - (Ci/LiY)R = 0,625
  - (Ci/LiY)M = 0,88
  - PIY = 0,76

Jika dibandingkan antara contoh pada Tabel 2.2 dengan contoh pada Tabel 2.3, maka dapat diambil kesimpulan bahwa air sungai yang diukur memenuhi baku mutu Y dan tidak memenuhi baku mutu X. Jadi bila nilai PI lebih kecil dari 1,0, maka sampel air tersebut memenuhi baku mutu termaksud, sedangkan bila lebih besar dari 1,0, sampel dinyatakan tidak memenuhi baku mutu.

Ditetapkan di : Jakarta pada tanggal : 10 Juli 2003

Menteri Negara Lingkungan Hidup,

**ttd**

**Nabiel Makarim, MPA, MSM**

**Salinan sesuai dengan aslinya**

**Deputi MENLH Bidang Kebijakan dan Kelembagaan Lingkungan Hidup,**

**Hoetomo, MPA.**
