# Supplementary figures and images for: The water quality of the Upper Citarum: Applying the overall index of pollution, Said-WQI, and pollution index methods
Source: Heliyon. 2025 Jan 8;11(2):e41690. doi: 10.1016/j.heliyon.2025.e41690 (PMC11787455; doi:10.1016/j.heliyon.2025.e41690)

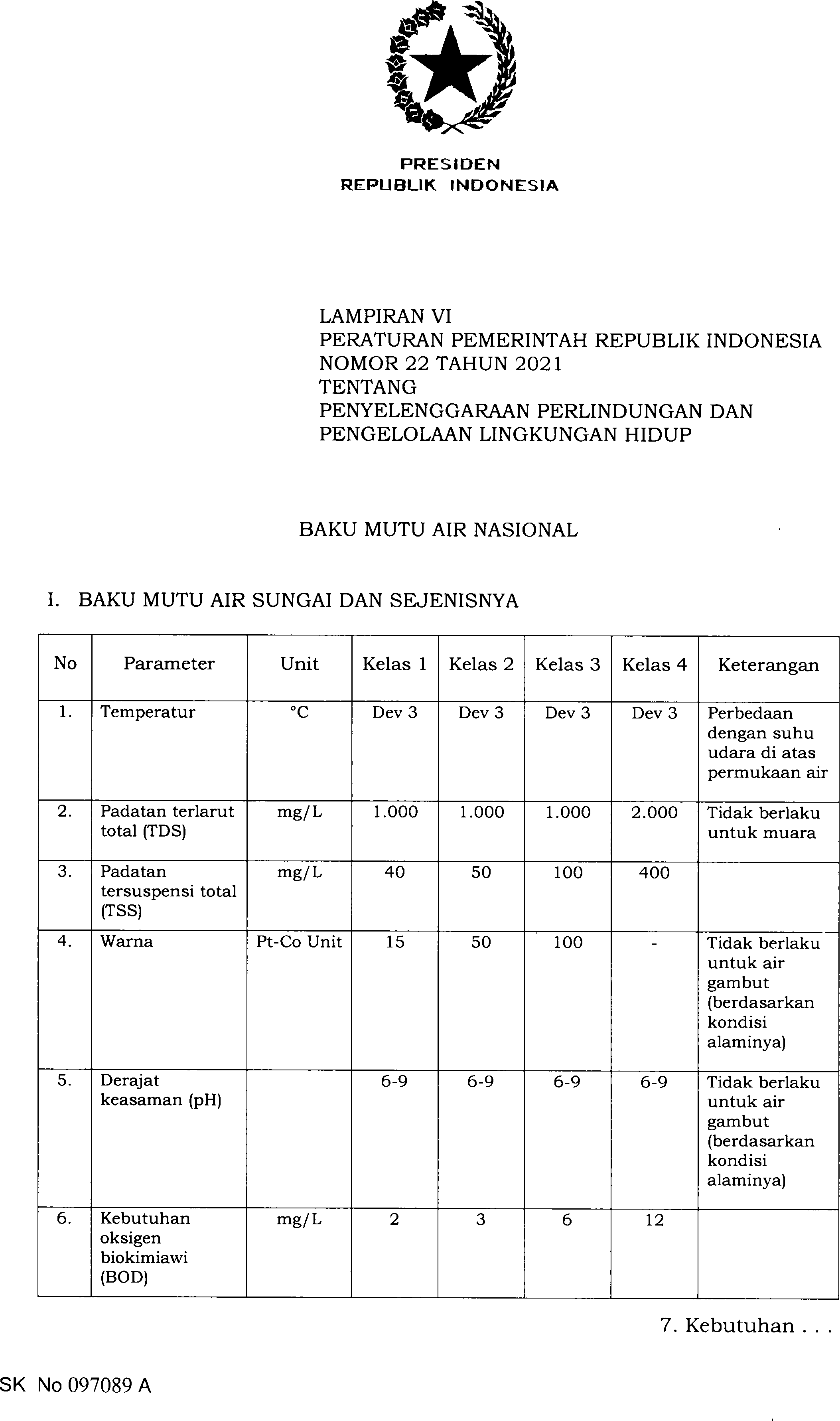


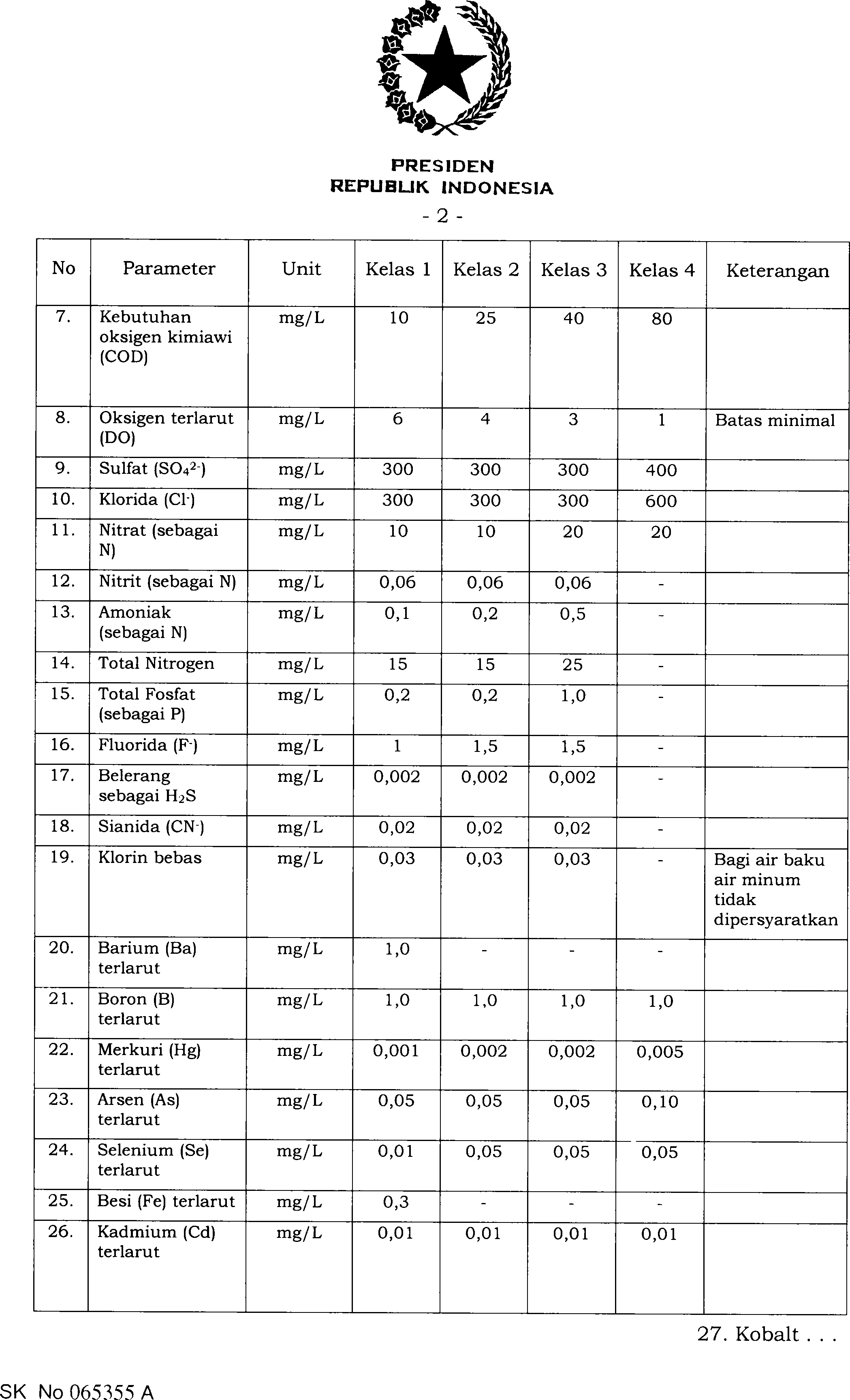


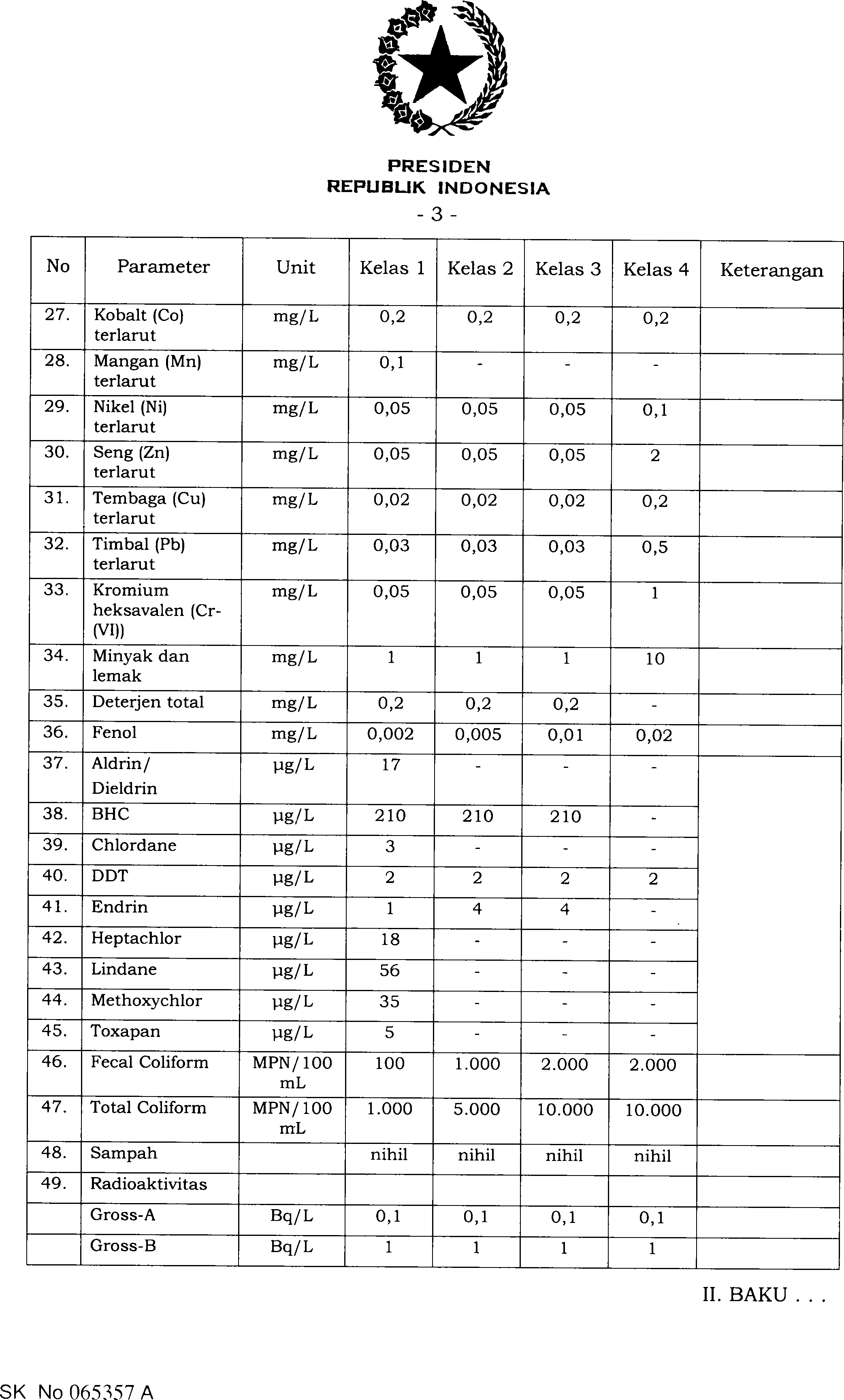


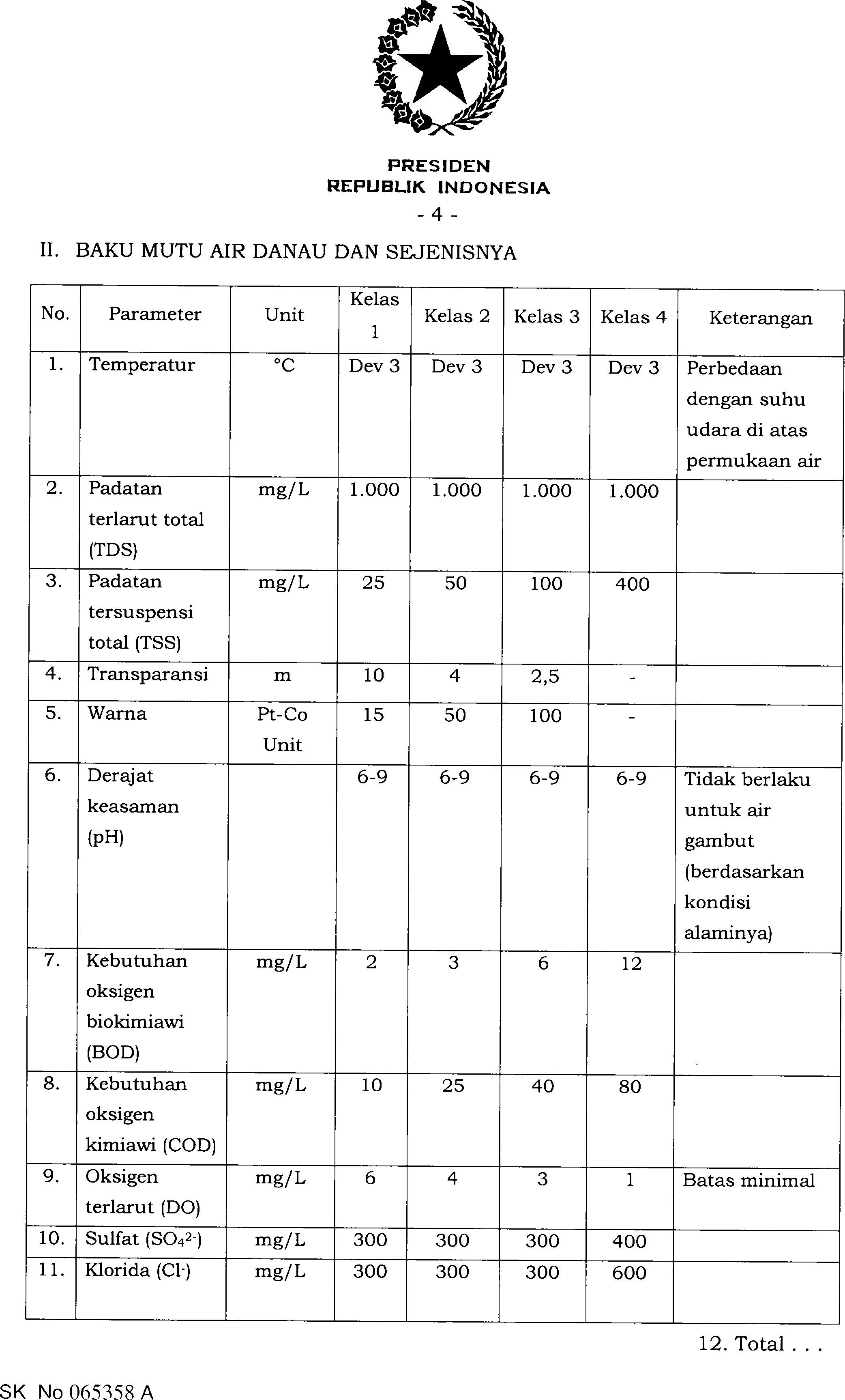


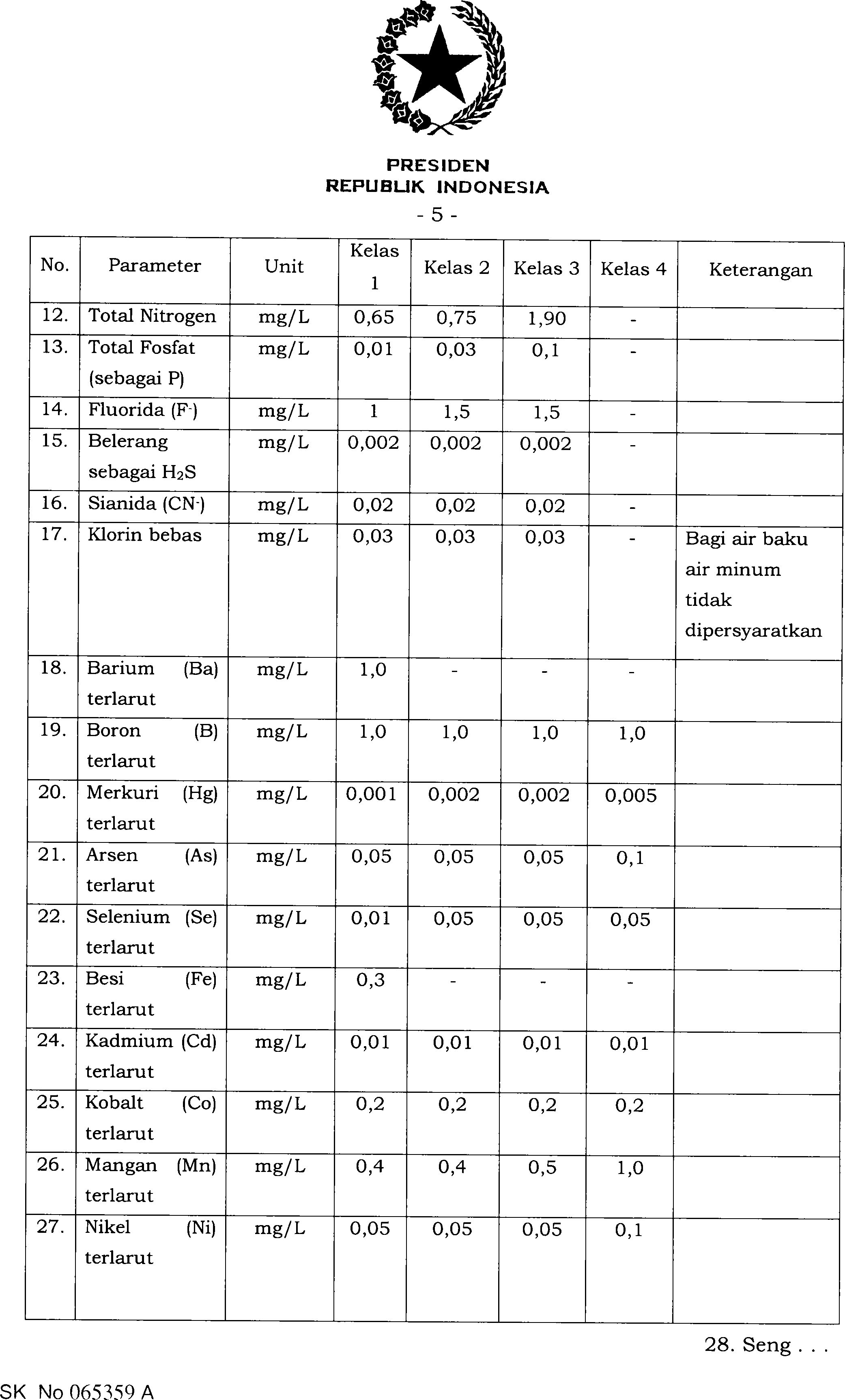


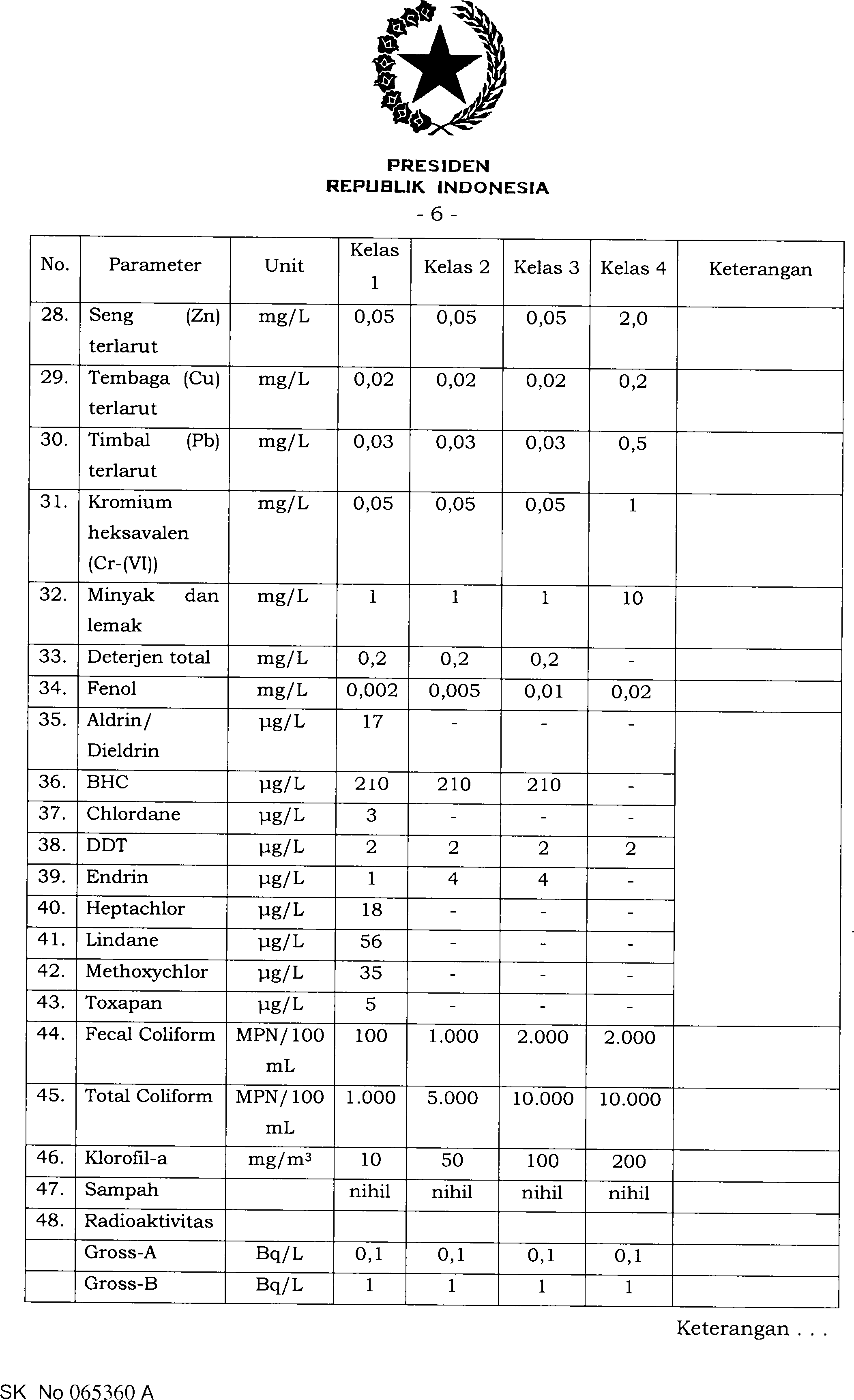


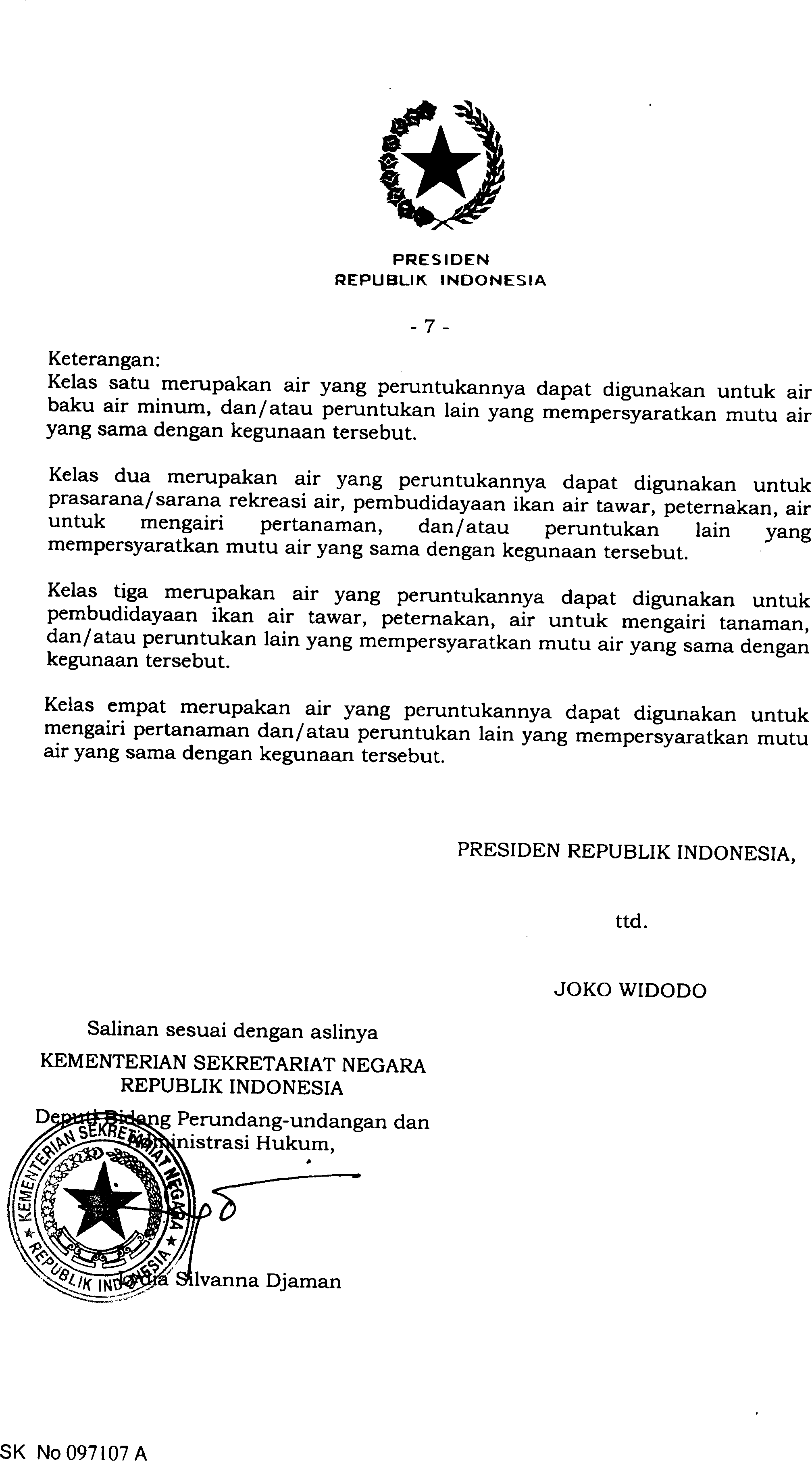

Supplement: Multimedia component 2 [file mmc2.docx]
